# Prevalence and influences of diabetes and prediabetes among adults living with HIV in Africa: a systematic review and meta‐analysis

**DOI:** 10.1002/jia2.26059

**Published:** 2023-03-16

**Authors:** Nasheeta Peer, Kim Anh Nguyen, Jillian Hill, Anne E. Sumner, Justin Cirhuza Cikomola, Jean Bisimwa Nachega, Andre‐Pascal Kengne

**Affiliations:** ^1^ Non‐communicable Diseases Research Unit South African Medical Research Council Durban and Cape Town South Africa; ^2^ Department of Medicine University of Cape Town Cape Town South Africa; ^3^ Section on Ethnicity and Health Diabetes Endocrinology and Obesity Branch National Institute of Diabetes and Digestive and Kidney Diseases National Institute of Health Bethesda Maryland USA; ^4^ National Institute on Minority Health and Health Disparities National Institutes of Health Bethesda Maryland USA; ^5^ Faculty of Medicine Université Catholique de Bukavu Bukavu the Democratic Republic of the Congo; ^6^ Division of Infectious Diseases Department of Medicine Stellenbosch University Faculty of Medicine and Health Sciences Cape Town South Africa; ^7^ Department of Epidemiology Infectious Diseases and Microbiology and Center for Global Health University of Pittsburgh Pittsburgh Pennsylvania USA; ^8^ Department of Epidemiology Johns Hopkins Bloomberg School of Public Health Baltimore Maryland USA; ^9^ Department of International Health Bloomberg School of Public Health Johns Hopkins University Baltimore Maryland USA

**Keywords:** Africa, ART, CD4 count, diabetes, prediabetes, HIV, risk factors, prevalence

## Abstract

**Introduction:**

In people living with human immunodeficiency virus (PLHIV), traditional cardiovascular risk factors, exposure to HIV per se and antiretroviral therapy (ART) are assumed to contribute to cardiometabolic diseases. Nevertheless, controversy exists on the relationship of HIV and ART with diabetes. To clarify the relationship between HIV and type 2 diabetes, this review determined, in PLHIV in Africa, diabetes and prediabetes prevalence, and the extent to which their relationship was modified by socio‐demographic characteristics, body mass index (BMI), diagnostic definitions used for diabetes and prediabetes, and HIV‐related characteristics, including CD4 count, and use and duration of ART.

**Methods:**

For this systematic review and meta‐analysis (PROSPERO registration CRD42021231547), a comprehensive search of major databases (PubMed‐MEDLINE, Scopus, Web of Science, Google Scholar and WHO Global Health Library) was conducted. Original research articles published between 2000 and 2021 in English and French were included, irrespective of study design, data collection techniques and diagnostic definitions used. Observational studies comprising at least 30 PLHIV and reporting on diabetes and/or prediabetes prevalence in Africa were included. Study‐specific estimates were pooled using random effects models to generate the overall prevalence for each diagnostic definition. Data analyses used R statistical software and “meta” package.

**Results:**

Of the 2614 records initially screened, 366 full‐text articles were assessed for eligibility and 61 were selected. In the systematic review, all studies were cross‐sectional by design and clinic‐based, except for five population‐based studies. Across studies included in the meta‐analysis, the proportion of men was 16–84%. Mean/median age was 30–62 years. Among 86,412 and 7976 participants, diabetes and prediabetes prevalence rates were 5.1% (95% CI: 4.3–5.9) and 15.1% (9.7–21.5). Self‐reported diabetes (3.5%) was lower than when combined with biochemical assessments (6.2%; 7.2%).

**Discussion:**

While not statistically significant, diabetes and prediabetes were higher with greater BMI, in older participants, urban residents and more recent publications. Diabetes and prediabetes were not significantly different by HIV‐related factors, including CD4 count and ART.

**Conclusions:**

Although HIV‐related factors did not modify prevalence, the diabetes burden in African PLHIV was considerable with suboptimal detection, and likely influenced by traditional risk factors. Furthermore, high prediabetes prevalence foreshadows substantial increases in future diabetes in African PLHIV.

## 1 INTRODUCTION

Despite a focus on infectious diseases in Africa, there is growing acknowledgement of the increasing burden of non‐communicable diseases (NCDs) as well as the double challenge of Africans experiencing both NCDs and infectious diseases. This is particularly true in people living with human immunodeficiency virus (PLHIV) following the successful rollout of highly active antiretroviral therapy (HAART), which has been accompanied by increased longevity [1, 2, 3]. The maturing of the HIV epidemic on the continent with ageing populations has subsequently led to exposure to NCDs, and a parallel increase in cardiovascular and cardiometabolic diseases.

The aetiology of cardiometabolic diseases in PLHIV is multifactorial. Together with traditional cardiovascular risk factors, such as ageing, obesity, unhealthy lifestyles and so on, exposure to HIV per se and HAART are assumed to contribute to cardiometabolic diseases [1, 2]. The use of HAART long‐term has been linked to dysregulation of glucose metabolism and dyslipidaemia, chronic systemic inflammation, endothelial dysfunction and an increase in cardiovascular disease (CVD) risk [1, 3–5].

Nevertheless, controversy exists, and debate is ongoing on the relationship of HIV and HAART with type 2 diabetes mellitus (hereafter referred to as diabetes); both increased risk and no difference have been described in European populations in high‐income countries [6]. In Africa, the global region with the greatest HIV burden (over 25 million individuals) [7], a meta‐analysis of a few heterogeneous studies published between 2008 and 2016, and with moderate‐to‐high risk of bias, revealed no significant association between prevalent diabetes and HIV or antiretroviral therapy (ART) [4]. In contrast, systematic reviews of studies prior to 2017 conducted in PLHIV globally have reported significant relationships between ART use and diabetes or prediabetes [5, 8]. Nevertheless, these reviews have highlighted the need for further research to explore the interactions between prediabetes and/or diabetes with ART in PLHIV [5].

Diabetes in PLHIV in Africa is poorly understood with insufficient information on the epidemiology and influences of this complex condition. This is of concern because of the increasing diabetes burden in general populations in Africa attributable to traditional risk factors [9], and likely a similar pattern in PLHIV. Moreover, unlike HIV, diabetes is inadequately detected and poorly controlled in Africa leading to a rising burden linked to premature death [9, 10]. Diabetes has the potential to threaten the advances in longevity achieved with the advent of ART in PLHIV in Africa [5]. Exploring and understanding the link between HIV and diabetes is important to maintain the advances made in the battle against HIV. Such information can inform strategies and interventions to effectively address comorbid diabetes in PLHIV [1, 2, 6].

This systematic review and meta‐analysis aimed to determine the pooled prevalence of diabetes and its precursor state, prediabetes, among adult PLHIV in Africa. Additionally, the meta‐analysis examined the magnitude of diabetes and prediabetes prevalence by socio‐demographic characteristics (age, gender and urban/rural residence), body mass index (BMI), diagnostic definitions used for diabetes and prediabetes, and HIV‐related characteristics (CD4 count, and use and duration of ART), among other predictive characteristics.

## 2 METHODS

This systematic review, focusing on the prevalence of prediabetes and diabetes in PLHIV in Africa (including North Africa), was registered in the PROSPERO registry for systematic reviews (registration number CRD42021231547) [11]. The systematic review and meta‐analyses were conducted in accordance with the Preferred Reporting Items for Systematic Reviews and Meta‐Analysis (PRISMA guidelines) [12].

### 2.1 Search strategy

A comprehensive electronic search was conducted across major databases, including PubMed‐MEDLINE, Scopus, Web of Science, Google Scholar and WHO Global Health Library. This was supplemented with manual scanning of reference lists of relevant articles and reviews. The search terms comprised combinations of MeSH terms, CINAHL headings and free words relating to prevalence, diabetes, prediabetes and HIV/AIDS. The search terms for PubMed‐MEDLINE are presented in Table S1 and were adapted accordingly for the other databases. The search was filtered for original research articles conducted in Africa and published from 01 January 2000 to 31 December 2021 in English and French languages.

### 2.2 Selection of eligible studies and diagnostic criteria

Observational studies (cross‐sectional, case–control and cohort studies) comprising at least 30 people, that reported on the prevalence of diabetes and/or prediabetes among adult PLHIV in Africa, were included. Studies reporting outcomes in pregnant women, children or type 1 diabetes only were excluded.

Criteria for diabetes included self‐report and/or biochemical testing using the following methods: oral glucose tolerance test (OGTT), fasting blood glucose (FBG) only, glycated haemoglobin (HbA1c) or random blood glucose (RBG). Prediabetes was determined on biochemical assessments only of the latter tests. Although the cut‐off points for diagnosing diabetes and prediabetes were not predefined, the biochemical cut‐points to diagnose diabetes across most studies were as follows: FBG ≥7 mmol/L and/or 2‐hour blood glucose ≥11.1 mmol/L; HbA1c ≥6.5%; and RBG ≥11.1 mmol/L. Prediabetes was generally diagnosed as follows: impaired fasting glycaemia (IFG): FBG: 6.1–6.9 mmol/L; impaired glucose tolerance (IGT): 2‐hour blood glucose: 7.8–11.0 mmol/L; HbA1c: 5.7–6.4% and RBG 7.8–11.0 mmol/L.

For the overall estimate of diabetes prevalence, each study was included once only irrespective of the number of criteria used for diagnosis. A tiered approach was used to include a single prevalence estimate as follows: (1) OGTT; (2) FBG; and (3) RBG. For example, if a study reported diabetes prevalence using both OGTT and FBG, the OGTT‐based diabetes prevalence was selected. Studies with self‐report diabetes estimates or data extracted from clinic folders were included. However, studies that utilized HbA1c only for the diagnosis of diabetes were excluded from the overall diabetes prevalence estimate because HbA1c has not yet been recommended for diabetes diagnostic purposes in African populations.

Similarly, for the overall estimate of prediabetes prevalence, the tiered approach was as follows: (1) OGTT; (2) IGT; (3) IFG; and (4) RBG. Two pooled prevalence estimates for prediabetes, with and without studies that used HbA1c only, were calculated. The studies that utilized HbA1c only were excluded from the sub‐group analyses.

### 2.3 Screening and data extraction

The studies were independently reviewed (KAN and NP) by title and abstract for eligibility, followed by an assessment of the relevant full texts. Disagreements were resolved by discussion and consensus or in consultation with a third investigator (APK). Relevant data for this review, extracted using a data extraction form designed for this review, included the following: (1) Manuscript details (author names and year of publication); (2) Study characteristics (country, study design, year of survey, study population, setting, sample size and sampling method); (3) Definitions (criteria used to define prediabetes or diabetes); and (4) Participant socio‐demographic and lifestyle characteristics (age, gender, smoking and alcohol use), HIV‐related factors (HIV stage, severity [CD4 count and viral load], duration of HIV diagnosis, ART regimen and duration of ART use) and comorbidities (obesity, hypertension, dyslipidaemia and co‐infections, such as tuberculosis and hepatitis).

### 2.4 Assessment of the methodological quality of included studies

The methodological quality of the included studies was evaluated using a checklist adapted from Hoy et al. [13] and used in previous systematic reviews [14]. The representativeness of the sample, the sampling technique, the response rate, the data collection method, the measurement tools, the case definitions and the statistical reporting were evaluated. Each of the nine questions were scored as low [1] or high (0) risk of bias. The total scores determined the risk of bias as follows: low: 7–9, moderate: 4–6 and high: 0–3.

The interrater disagreement was resolved by consensus or in consultation with a third investigator (APK). The precision (C) or margin of error was estimated for each included study, considering the sample size (SS) and the observed prevalence (p) of diabetes/prediabetes from the formula SS = Z2_p_(1–p)/C2, where Z is the z‐value fixed at 1.96 across studies (corresponding to the 95% confidence interval). The desirable margin of error was 5% (0.05) or lower.

### 2.5 Data synthesis and analyses

Data analyses were conducted using the R statistical software and the “meta” package. For each included study, the unadjusted prevalence of diabetes and prediabetes were estimated overall and across the major sub‐groups of interest. The study‐specific estimates were pooled using random effects models to generate the overall prevalence of diabetes and prediabetes for each diagnostic definition. The variance of the raw prevalence was stabilized using the Freeman–Tukey double arc‐sine transformation before pooling the data to minimize the effect of extreme prevalence on the overall estimates. Data are presented as prevalence (%) and 95% confidence intervals (CI). A *p*‐value <0.05 described statistically significant differences in findings within each diagnostic criterion overall, and by sub‐group analyses.

Heterogeneity among studies was assessed using *I* ^2^, Cochran's *Q* and *H* statistics. *I* ^2^ values of <50% represented low heterogeneity and >75% described high heterogeneity. Potential sources of heterogeneity were explored by comparing the prevalence of diabetes and prediabetes between sub‐groups of interest. These comparisons used the *Q*‐test based on the Analysis of the Variance. Differences in major characteristics, such as study design, study populations, and diagnostic criteria and cut points for diabetes and prediabetes, were used to define sub‐groups of interest, for example discrete categories (gender, setting, year of publication, diagnostic criteria and ART use) or by using median values of the summary estimates for continuous characteristics (age, BMI, sample size and ART duration).

The presence of publication bias was assessed using the funnel plots. This was supplemented by formal statistical assessments using the Egger test of bias [15]. A *p*‐value <0.05 illustrated a significant asymmetry of the funnel plot and evidence of publication bias. The Duval and Tweedie trim‐and‐fill was used to adjust estimates for the effects of publication bias.

Ethical approval was not required as this was secondary analyses of published data.

## 3 RESULTS

### 3.1 The review processes and data extraction

**Figure 1:**
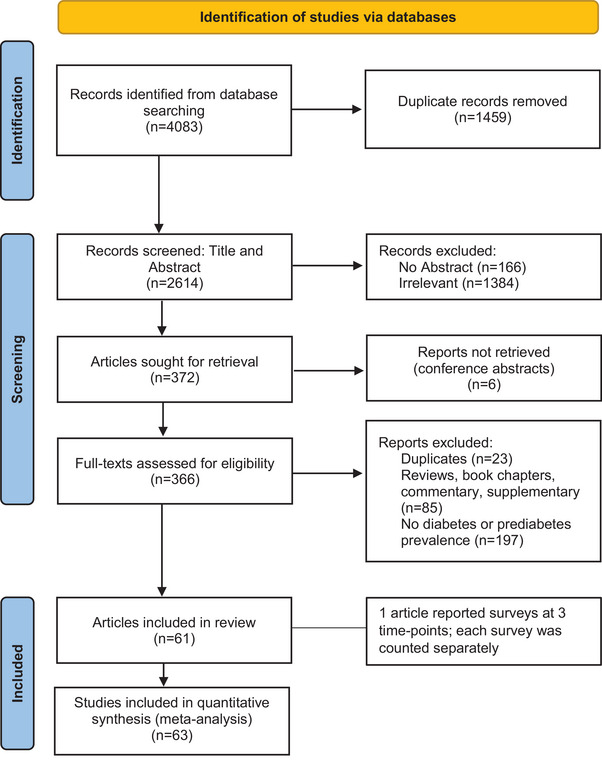
Preferred Reporting Items for Systematic Reviews and Meta‐Analysis (PRISMA) diagram.

After duplicate removals from the 4083 records identified, titles and abstracts of 2614 records were screened, and 366 full‐text articles were assessed for eligibility (Figure 1). Of these, 61 fulfilled the eligibility criteria and were included in this review. One article [16] reported surveys at three time points which were counted separately, making a total of 63 studies included in the meta‐analysis.

All relevant HIV‐related factors (HIV staging and viral load) and co‐morbidities (hypertension, dyslipidaemia and co‐infections) were not extracted as planned because of the lack of such data or an inadequate number of studies reporting the requisite data for meaningful analyses.

### 3.2 Methodological quality of the included studies

**Figure 2:**
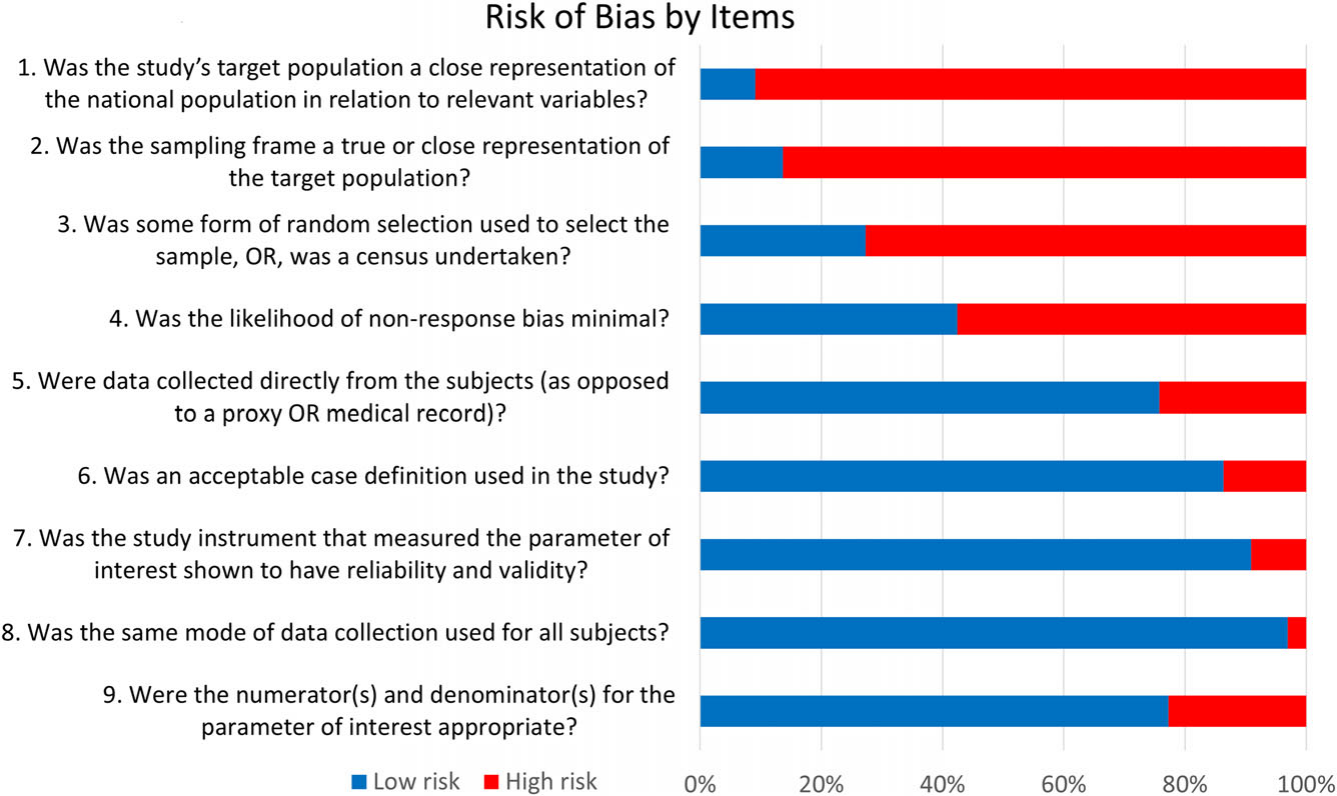
Risk of bias assessments for the included studies.

The risk of bias assessment for the included studies is summarized in Figure 2. Eight studies had a low risk of bias and 55 studies had a moderate risk of bias. Among the latter, 33 studies involved less than 500 participants, while 16 studies reported using some form of random selection approach to select participants.

### 3.3 General characteristics of the included studies

Studies were published between 2008 and 2021 (Table 1). One study was published before 2010 [17], nine were published from 2011 to 2015 [18, 19, 20, 21, 22, 23, 24, 25, 26] and 5–10 studies were published yearly thereafter. The highest number of included studies were from South Africa (*n* = 18) [16, 18, 34–24, 27, 39–33], followed by Ethiopia (*n* = 8) [26, 40–46] and Tanzania (*n* = 7) [19, 22, 25, 47–50], with four studies each from Cameroon [19, 51–53], Ghana [21, 54–56], Kenya [17, 57–59], Malawi [60, 61, 62, 63] and Uganda [64, 65, 66, 67].

**Table 1:** Summary of the characteristics and methodological quality of the included studies

Authors	Published year	Country	Area	Study site	Study type	Study period	Sampling	*N*	Male %	Mean/median age	Selection criteria	Quality grade (risk)	Margin of error
Manuthu et al. [17]	2008	Kenya	Urban	Clinic‐based	Cross‐sectional	2006	Non‐random	295	ART naïve: 44; on ART: 40	ART naïve: 36.5; on ART: 39.4	Adult PLHIV on ART ≥4 weeks and not changing ART regimen in the past year, and without history of diabetes or taking lipid‐lowering agents.	Moderate	0.045
Dave et al. [18]	2011	South Africa	Urban	Clinic‐based	Cross‐sectional	NR	Non‐random	849	23	−	PLHIV on first‐line ART regimen (d4T, 3TC and efavirenz or nevirapine) >6 months and ART naïve, and without history of diabetes or impair glucose tolerance.	Moderate	0.017
Nagu et al. [19]	2012	Tanzania	Urban	Clinic‐based	Cross‐sectional	2004–2009	Non‐random	41,891	29	36 (10)	PLHIV >15 years, ART naïve (83% >18 years)	Moderate	0.002
Ngatchou et al. [20]	2013	Cameroon	Urban	Clinic‐based	Cross‐sectional	2009–2010	Unspecified	108	26	39 (11)	PLHIV ≥18 years, ART naive	Moderate	0.082
Ngala et al. [21]	2013	Ghana	Urban	Clinic‐based	Cross‐sectional	2009–2010	Unspecified	164	42	38.2 (0.65)	PLHIV ≥18 years, on ART ≥6 months, without previous diabetes, hypertension or family history of diabetes, hypertension.	Moderate	0.038
Kagaruki et al. [22]	2014	Tanzania	Urban; rural	Clinic‐based	Cross‐sectional	2011–2012	Non‐random	671	30	38.7 (10.1	PLHIV ≥18 years	Low	0.015
Sawadogo et al. [23]	2014	Burkina Faso	Urban	Clinic‐based	Cross‐sectional	2011	Random	400	29	41.4 (8.8)	PLHIV ≥18 years, on ART ≥6 months	Moderate	0.011
Rabkin et al. [24]	2015	South Africa	Urban	Clinic‐based	Cross‐sectional	2014	Non‐random	175	26	45.4 (8.8)	PLHIV ≥30 years on ART ≥1 year	Moderate	0.029
Maganga et al. [25]	2015	Tanzania	Urban	Clinic‐based	Cross‐sectional	2012–2013	Non‐random	301	ART naïve: 41; on ART: 23	ART naïve: 37 (32–44); on ART: 40 (38–47)	PLHIV ≥18 years ART naive, or on ART ≥2 years	Moderate	0.013
Mohammed et al. [26]	2015	Ethiopia	Urban; rural	Clinic‐based	Cross‐sectional	2014	Non‐random	393	33	37.9 (11.2)	PLHIV ≥18 years	Moderate	0.024
Divala et al. [60]	2016	Malawi	Urban; rural	Clinic‐based	Cross‐sectional	2014	Non‐random	952	28	43 (10.2)	PLHIV ≥18 years	Moderate	0.013
Mugisha et al. [64]	2016	Uganda	Urban; rural	Population‐based	Cross‐sectional	2012–2013	Non‐random	244	40	57	PLHIV ≥50 years	Moderate	0.03
Isa et al. [76]	2016	Nigeria	Urban	Clinic‐based	Cross‐sectional	2011–2013	Non‐random	2632	35	37.4 (9.7)	PLHIV ≥18 years on ART	Moderate	0.006
Rhee et al. [51]	2016	Cameroon	Urban	Clinic‐based	Cross‐sectional	2014	Non‐random	500	27	42.5 (36.5–49.5)	PLHIV ≥16 years on ART (>80% age ≥18 years), and without diabetes history	Moderate	0.017
Levitt et al. [27]	2016	South Africa	Urban; rural	Population‐based	Cross‐sectional	2008–2010	Non‐random	940	24	33.6 (8.77)	PLHIV ≥18 years, without diabetes history	Moderate	0.014
Magodoro et al. [1]	2016	Zimbabwe	Urban	Clinic‐based	Cross‐sectional	2015	Non‐random	1400	31	42 (36–50)	PLHIV ≥18 years on ART	Moderate	0.009
Abebe et al. [46]	2016	Ethiopia	Urban; rural	Clinic‐based	Cross‐sectional	2013–2014	Random	462	31	ART naïve: 34.6 (9.9); on ART: 37.5 (9.2)	PLHIV ≥15 years (93% ≥18 years)	Moderate	0.025
Sinxadi et al. [28]	2016	South Africa	Urban	Clinic‐based	Cross‐sectional	2007–2008	Non‐random	107	27	38 (31–45)	PLHIV ≥18 years on EFV for ≥6 months and adherence to ART, without previous diabetes	Moderate	0.026
Traoré et al. [77]	2016	Morocco	Urban	Clinic‐based	Retrospective	−	Non‐random	1800	−	−	PLHIV treated at the Infectious Diseases out‐patient department of the University Hospital Center of Casablanca (Ibn Rochd), Morocco	Moderate	0.008
Ekrikpo et al. [78]	2017	Nigeria	Urban	Clinic‐based	Cross‐sectional	2002–2016	Non‐random	1818	40	34.3 (9.9)	PLHIV ≥18 years, ART naive	Moderate	0.011
Noumegni et al. [52]	2017	Cameroon	Urban	Clinic‐based	Cross‐sectional	2015–2016	Non‐random	452	20	44.4 (9.8)	PLHIV ≥18 years, ART naïve or on ART; without CVD history	Moderate	0.014
PrayGod et al. [50]	2017	Tanzania	Urban	Clinic‐based	Cross‐sectional	2015	Non‐random	273	35	38.9 (9.7)	PLHIV ≥18 years on ART for 2–3 years	Moderate	0.014
Gaziano et al. [29]	2017	South Africa	Rural	Population‐based	Cross‐sectional	2014–2015	Random	4576	46	61.7 (13.1)	PLHIV ≥40 years	Moderate	0.015
Van Heerden et al. [30]	2017	South Africa	Rural	Population‐based	Cross‐sectional	2011–2012	Non‐random	189	19	−	PLHIV ≥18 years	Moderate	0.02
Shankalala et al. [79]	2017	Zambia	Urban	Clinic‐based	Cross‐sectional	2015	Non‐random	270	31	46 (38–51)	PLHIV ≥18 years on ART ≥24 months	Moderate	0.039
Kazooba et al. [65]	2017	Uganda	Rural	Clinic‐based	Cross‐sectional	2014	Non‐random	1024	35	44.8 (8)	PLHIV ≥18 years	Moderate	0.011
Labhardt et al. [31]	2017	South Africa	Rural	Clinic‐based	Cross‐sectional	2014	Non‐random	1166	34	44.4 (35.3–54.4)	PLHIV ≥16 years, on NNRTI‐based first‐line ≥6 months	Low	0.008
Pfaff et al. [61]	2018	Malawi	Urban	Clinic‐based	Cross‐sectional	2015–2017	Non‐random	2979	−	−	PLHIV ≥18 years	Moderate	0.003
Ngu et al. [53]	2018	Cameroon	Urban	Clinic‐based	Cross‐sectional	−	Non‐random	311	16	43.4 (10.6)	PLHIV ≥21 years	Moderate	0.035
Mathabire Rücker et al. [62]	2018	Malawi	Urban	Clinic‐based	Cross‐sectional	2015–2016	Non‐random	379	26	47 (42–52)	PLHIV ≥ 30 years on ART ≥10 years	Moderate	0.025
Kansiime et al. [66]	2018	Uganda	Urban	Clinic‐based	Cross‐sectional	2017	Non‐random	387	34	42 (20–75)	PLHIV ≥18 years on ART ≥2 months	Moderate	0.021
Ataro et al. [45]	2018	Ethiopia	Urban	Clinic‐based	Cross‐sectional	2017	Non‐random	425	30	39.7 (8.9)	PLHIV ≥years on ART ≥6 months	Moderate	0.024
Rabkin et al. [68]	2018	Swaziland	Urban	Clinic‐based	Cross‐sectional	2015–2016	Non‐random	1826	38	47 (40–82)	PLHIV ≥40 years	Moderate	0.009
Katoto et al. [80]	2018	DRC	Urban	Clinic‐based	Cross‐sectional	2016	Non‐random	495	38	43 (36–51)	PLHIV ≥18 years	Moderate	0.027
Osoti et al. [57]	2018	Kenya	Urban; rural	Clinic‐based	Cross‐sectional	2014	Non‐random	300	36	40 (33–46)	PLHIV ≥18 years	Moderate	0.031
Fiseha and Belete [40]	2019	Ethiopia	Urban; peri‐urban	Clinic‐based	Cross‐sectional	2018	Unspecified	408	33	37 (10)	PLHIV ≥ 18 years on ART ≥12 months	Low	0.027
Muchira et al. [67]	2019	Uganda	Urban; rural	Clinic‐based	Cross‐sectional	NR	Non‐random	118	51.7	51.3 (7.1)	PLHIV ≥40 years on ART ≥3 years	Moderate	0.071
Faurholt‐Jepsen et al. [41]	2019	Ethiopia	Urban	Clinic‐based	Cross‐sectional	2010–2012	Non‐random	332	33	32.9 (8.8)	PLHIV ≥ 18 years, ART naïve	Moderate	0.036
Juma et al. [58]	2019	Kenya	Rural	Clinic‐based	Cross‐sectional	2013–2015	Non‐random	1502	31	30 (31–48)	PLHIV ≥18 years, ART naïve or on ART	Moderate	0.003
Hyle et al. [32]	2019	South Africa	Urban	Clinic‐based	Cross‐sectional	2015–2016	Non‐random	458	22	38 (33–44)	PLHIV ≥21 years, on ART	Moderate	0.021
Nguyen et al. [33]	2019	South Africa	Urban; rural	Clinic‐based	Cross‐sectional	2014–2015	Random	748	21	38 (35–42)	PLHIV ≥18 years, ART naïve or on ART	Low	0.013
Nkinda et al. [48]	2019	Tanzania	Urban	Clinic‐based	Cross‐sectional	2018	Non‐random	240	25	47 (10)	PLHIV ≥18 years, on first‐line ART regimen	Moderate	0.039
Appiah et al. [54]	2019	Ghana	Urban	Clinic‐based	Cross‐sectional	2013–2014	Non‐random	345	28	41 (11)	PLHIV ≥18 years	Moderate	0.021
Zungu et al. [34]	2019	South Africa	Urban; rural	Clinic‐based	Cross‐sectional	2015–2016	Random	2648	23	−	PLHIV educators ≥18 years	Moderate	0.009
Sogbanmu et al. [35]	2019	South Africa	Urban	Clinic‐based	Cross‐sectional	2016–2017	Random	335	31	−	PLHIV adults, ART naive	Moderate	0.026
Jeremiah et al. [47]	2020	Tanzania	Urban	Clinic‐based	Cross‐sectional	2016–2017	Non‐random	ART naïve: 956; on ART: 336	39	41 (11)	PLHIV ≥ 18 years, ART naïve or on ART	Moderate	0.009
Kato et al. [49]	2020	Tanzania	Urban	Clinic‐based	Cross‐sectional	2017	Non‐random	612	30	47 (42–52)	PLHIV ≥18 years: ART naïve or on ART ≥5 years	Moderate	0.021
Gebrie et al. [42]	2020	Ethiopia	Urban; rural	Clinic‐based	Cross‐sectional	2019	Random	407	40	38.6 (10.29)	PLHIV ≥18 years on ART ≥6 months	Low	0.027
Duguma et al. [43]	2020	Ethiopia	Urban; rural	Clinic‐based	Cross‐sectional	2019	Random	271	37	38.5 (8.98)	PLHIV ≥18 years on ART ≥3 months	Moderate	0.038
Woldesemayat [44]	2020	Ethiopia	Urban	Clinic‐based	Cross‐sectional	2016	Random	382	39	35 (10)	PLHIV ≥18 years on ART	Low	0.014
Singano et al. [63]	2021	Malawi	Urban	Clinic‐based	Cross‐sectional	2018	Non‐random	1316	30	44 (38–53)	PLHIV ≥18 years on ART ≥6 months	Moderate	0.008
Umar and Naidoo [36]	2021	South Africa	Urban	Clinic‐based	Cross‐sectional	2005–2009	Random	1203	34	29–48 (60%)	PLHIV ≥18 years on ART ≥6 months	Moderate	0.016
Chiwandire et al. [16]	2021	South Africa	Urban; rural	Clinic‐based	Cross‐sectional	2005	Random	978	32	−	PLHIV ≥25 years	Moderate	0.012
Chiwandire et al. [16]	2021	South Africa	Urban; rural	Clinic‐based	Cross‐sectional	2008	Random	1023	31	−	PLHIV ≥25 years	Moderate	0.011
Chiwandire et al. [16]	2021	South Africa	Urban; rural	Clinic‐based	Cross‐sectional	2017	Random	2483	29	−	PLHIV ≥25 years	Moderate	0.008
Rajagopaul and Naidoo [37]	2021	South Africa	Urban	Clinic‐based	Cross‐sectional	2017	Non‐random	301	37.5	41.6 (11)	PLHIV ≥18 years, on ART	Moderate	0.016
Kubiak et al. [38]	2021	South Africa	Urban	Clinic‐based	Cross‐sectional	2017–2019	Non‐random	1207	44.4	31.3 (9.5)	PLHIV ≥18 years, ART naive	Moderate	0.008
Njoroge et al. [59]	2021	Kenya	Urban; rural	Clinic‐based	Cross‐sectional	2018	Random	600	36.2	46.8 (41.6–53.1)	PLHIV >35 years old, on ART for at least 5 years	Low	0.017
Chezac et al. [81]	2021	Zimbawe	Urban	Clinic‐based	Cross‐sectional	2010	Non‐random	203	37	−	PLHIV on ART enrolled at Chitungwiza Central Hospital's Opportunistic Infections Clinic in 2010	Moderate	0.034
Sanuade et al. [55]	2021	Ghana	Urban	Clinic‐based	Cross‐sectional	–	Random	525	84	33.6 (5.0)	PLHIV ≥18 years	Moderate	0.031
Sarfo et al. [56]	2021	Ghana	Urban; rural	Clinic‐based	Cross‐sectional	–	Non‐random	502	24.8	44	PLHIV ≥30 years	Moderate	0.022
Hird et al. [39]	2021	South Africa	Urban	Population‐based	Cross‐sectional	2014	Random	487	−	−	PLHIV ≥18 years	Low	0.01
Hema et al. [82]	2021	Burkina Faso	Urban	Clinic‐based	Cross‐sectional	2018	Non‐random	4259	26.1	45 (38–52)	PLHIV >18 years old on ART	Moderate	0.008

Abbreviation: PLHIV, people living with HIV.

All studies were cross‐sectional and clinic‐based, except five studies that were population‐based (four from South Africa and one from Uganda). Forty studies were conducted in urban settings only, five in rural settings only [29–31, 58, 65] and 18 in both urban and rural settings. Most studies (*n* = 44) recruited unselected samples, while 16 studies used random sampling techniques; three studies did not specify the sampling technique.

Most studies (*n* = 44) had less than 1000 participants and 11 studies had between 1000 and 2000 participants; the sample sizes ranged from 107 to 41,891 participants. The proportion of males in the studies ranged from 16% to 84%. The mean/median age ranged from 30 to 62 years. Most studies were conducted in ≥18‐year‐old adults but a few focused on older adults (≥30 years: *n* = 3 [24, 56, 62]; ≥40 years: *n* = 3 [29, 67, 68]; ≥ 50 years: *n* = 1 [64]).

### 3.4 Biochemical tests utilized in included studies

Among the 63 included studies, 35 defined diabetes using biochemical criteria and/or a self‐reported diagnosis, 20 defined diabetes using biochemical criteria only and 11 studies described self‐reported diabetes only (Table S2). The most common biochemical tests used were OGTT and FBG (Table S2). Seven studies used HbA1c alone to diagnose diabetes and were excluded from the overall diabetes prevalence estimate [24, 31, 38, 51, 59, 62, 68] because HbA1c has been shown to underperform in African populations [69]. Estimates from 56 studies were pooled to determine the overall diabetes prevalence. However, five studies reported diabetes prevalence in sub‐groups only and were counted as separate studies. These included four that determined diabetes prevalence separately in ART naïve and ART users [18, 25, 49, 57], and a single study that described diabetes prevalence in ART naïve, first‐line ART users and second‐line ART users [18]. Thus, a total of 62 studies were pooled to derive the overall diabetes prevalence estimate (Table 2).

**Table 2:** Summary statistics for the meta‐analyses of the prevalence studies on diabetes in people with HIV in Africa using random effects model and double‐arcsine transformations

**Group**	**Sub‐group**	**Criteria**	***N* studies**	***N* participants**	***N* cases**	**Prevalence (95 CI)**	***H* (95 CI)**	***I* ^2^ (95 CI)**	***p*‐heterogeneity**	***p*‐dif criteria**	***p*‐diff sub‐groups**	***p*‐Egger test**
**Overall**		Any criteria	62	86,412	3559	5.05 [4.27–5.89]	4.59 [4.25–4.94]	95.2 [94.5–95.9]	<0.001	0.937		0.002
	Biochemical criteria only									0.165		
		OGTT	13	5213	217	3.31 [1.93–5.02]	2.95 [2.37–3.67]	88.5 [82.1–92.6]	<0.001			0.390
		FBG	9	2405	229	7.89 [4.56–12.00]	3.29 [2.56–4.23]	90.7 [84.7–94.4]	<0.001			0.401
		RBG	2	459	37	6.30 [1.40–19.46]	4.33 [2.47–7.58]	94.7 [83.6–98.3]	<0.001			–
		HbA1c	7	4155	287	5.06 [1.70–9.99]	5.82 [4.74–7.14]	97.0 [95.5–98.0]	<0.001			0.632
		Mixed criteria	4	6750	293	3.08 [0.00–12.30]	14.70 [12.57–17.18]	99.5 [99.4–99.7]	<0.001			0.541
	Self‐report only									0.274		
		Self‐report	13	14,797	470	3.48 [2.17–5.07]	4.63 [3.91–5.48]	95.3 [93.5–96.7]	<0.001			0.264
		Patient folder	7	5453	332	5.00 [2.85–7.71]	3.94 [3.03–5.11]	93.5 [89.1–96.2]	<0.001			0.417
	Biochemical criteria and/or self‐report									<0.001		
		OGTT	5	1932	138	6.18 [2.49–11.31]	3.96 [2.89–5.43]	93.6 [88.0–96.6]	<0.001			0.846
		FBG	14	10,001	702	7.16 [5.31–9.26]	3.49 [2.88–4.22]	91.8 [87.9–94.4]	<0.001			0.683
		RBG	1	1502	7	0.47 [0.17–0.89]	−	−	−			−
		HbA1c	4	2991	153	4.86 [2.40–8.10]	3.18 [2.11–4.78]	90.1 [77.6–95.6]	<0.001			0.998
		Mixed criteria	13	55,145	1740	4.15 [3.05–5.41]	4.52 [3.81–5.36]	95.1 [93.1–96.5]	<0.001			0.215
**Age**												
Median age, 41 years old	≥39 years	Any criteria	25	18,378	1053	5.96 [4.51–7.60]	4.20 [3.70–4.77]	94.3 [92.7–95.6]	<0.001	0.002	0.129	0.448
	Biochemical criteria only									0.375		
		OGTT	2	1440	125	11.99 [3.71–24.02]	3.74 [2.05–6.83]	92.8 [76.1–97.9]	<0.001		0.016	0.523
		FBG	6	1580	131	7.67 [3.70–12.87]	3.30 [2.41–4.52]	90.8 [82.8–95.1]	<0.001		0.891	0.986
		RBG	1	270	33	12.22 [8.56–16.42]	−	−	−		−	
		HbA1c	5	2461	254	6.91 [2.75–12.71]	4.40 [3.27–5.92]	94.8 [90.7–97.1]	<0.001		0.019	0.176
		Mixed criteria	3	3771	280	4.48 [0.00–20.26]	15.84 [13.22–18.98]	99.6 [99.4–99.7]	<0.001		−	0.915
	Self‐report								0.091			
		Self‐report	7	6561	209	3.78 [2.39–5.47]	2.99 [2.21–4.06]	88.8 [79.5–93.9]	<0.001		0.686	0.180
		Patient folder	2	1535	37	2.40 [1.64–3.28]	1.04 [–]	8.3 [–]	0.296		0.774	−
	Biochemical criteria and/or self‐report									0.346		
		OGTT	2	546	54	6.66 [0.00–30.20]	7.54 [5.01–11.36]	98.2 [96.0–99.2]	<0.001		0.919	
		FBG	7	7057	487	6.89 [3.84–10.72]	4.74 [3.76–5.99]	95.6 [92.9–97.2]	<0.001		0.802	0.997
		RBG	0	−	−	−	−	−	−			
		HbA1c	3	2707	129	3.96 [1.52–7.46]	3.43 [2.14–5.50]	91.5 [78.2–96.7]	<0.001		0.050	0.736
		Mixed criteria	8	4359	165	3.89 [2.88–5.04]	1.71 [1.17–2.49]	65.7 [27.1–83.9]	0.004		0.328	0.296
	<39 years	Any criteria	24	55,455	1868	4.50 [3.45–5.68]	3.99 [3.49–4.56]	93.7 [91.8–95.2]	<0.001	0.131		0.002
	Biochemical criteria only									0.095		
		OGTT	7	2142	54	2.33 [1.60–3.18]	1.13 [1.00–1.68]	21.2 [0.0–64.5]	0.267			0.883
		FBG	3	825	98	8.28 [2.44–16.99]	3.42 [2.13–5.48]	91.4 [78.0–96.7]	<0.001			0.070
		RBG	0	−	−	−	−	−	−			
		HbA1c	1	1207	26	2.15 [1.40–3.06]	−	−	−			
		Mixed criteria	0	−	−	−	−	−	−			
	Self‐report									0.148		
		Self‐report	3	2147	75	3.48 [2.74–4.30]	1.00 [1.00–3.10]	0.0 [0.0–89.6]	0.699			0.327
		Patient folder	1	382	8	2.09 [0.86–3.81]	−	−	−			
	Biochemical criteria and/or self‐report									<0.001		
		OGTT	3	1386	84	5.90 [4.18–7.89]	1.41 [1.00–2.62]	49.7 [0.0–85.4]	0.136			0.815
		FBG	6	2482	178	7.42 [5.41–9.72]	2.05 [1.37–3.07]	76.2 [46.6–89.4]	0.001			0.034
		RBG	1	1502	7	0.47 [0.17–0.89]	−	−	−			
		HbA1c	1	284	24	8.45 [5.47–11.99]	−	−	−			
		Mixed criteria	5	47,131	1490	5.14 [3.32–7.32]	4.88 [3.70–6.45]	95.8 [92.7–97.6]	<0.001			0.165
**Gender**												
	Men	Any criteria	22	8142	423	4.85 [3.59–6.29]	2.67 [2.24–3.18]	86.0 [80.0–90.1]	<0.001	0.223	0.524	0.994
	Biochemical criteria only									<0.001		
		OGTT	2	540	8	1.43 [0.53–2.68]	1.00	00.0	0.923		0.278	
		FBG	2	170	15	8.54 [4.64–13.38]	1.00	00.0	0.325		0.249	
		RBG	0	−	−	−	−	−	−			
		HbA1c	2	581	13	1.85 [0.79–3.25]	1.00	00.0	0.737		0.625	
		Mixed criteria	0	−	−	−	−	−	−			
	Self‐report									0.798		
		Self‐report	6	2428	92	3.36 [1.89–5.22]	2.20 [1.49–3.25]	79.3 [54.8–90.5]	<0.001		0.990	0.647
		Patient folder	3	567	19	3.71 [0.95–7.95]	2.03 [1.12–3.69]	75.8 [20.3–92.7]	0.016		0.589	0.308
	Biochemical criteria and/or self‐report									0.115		
		OGTT	1	157	10	6.37 [3.01–10.80]	−	−	−		0.889	
		FBG	5	1873	164	8.05 [4.95–11.81]	2.30 [1.51–3.51]	81.1 [56.1–91.9]	<0.001		0.119	0.763
		RBG	0	−	−	−	−	−	−			
		HbA1c	0	−	−	−	−	−	−			
		Mixed criteria	6	3104	141	4.28 [2.59–6.35]	2.45 [1.69–3.55]	83.4 [65.2–92.1]	<0.001		0.958	0.963
	Women	Any criteria	22	17,846	827	4.47 [3.66–5.36]	2.60 [2.17–3.11]	85.2 [78.8–89.7]	<0.001	0.324		0.946
	Biochemical criteria only									0.009		
		OGTT	2	1298	32	2.48 [1.40–3.85]	1.41 [–]	49.8 [–]	0.158			–
		FBG	2	419	26	6.14 [3.99–8.68]	1.00 [–]	0.0 [–]	0.493			–
		RBG	0	−	−	−	−	−	−			
		HbA1c	2	801	20	2.85 [0.84–5.87]	1.61 [1.00–3.35]	61.5 [0.0–91.1]	0.107			–
		Mixed criteria	0	−	−	−	−	−	−			
	Self‐report									0.636		
		Self‐report	6	6370	262	3.46 [2.16–5.05]	3.07 [2.21–4.26]	89.4 [79.5–94.5]	<0.001			0.179
		Patient folder	3	1178	32	2.80 [1.25–4.88]	1.71 [1.00–3.18]	65.6 [0.0–90.1]	0.054			0.663
	Biochemical criteria and/or self‐report									0.297		
		OGTT	1	591	37	6.26 [4.44–8.37]						
		FBG	5	4673	280	5.82 [4.41–7.40]	1.70 [1.05–2.75]	65.3 [9.1–86.7]	0.021			0.957
		RBG	0	−	−	−	−	−	−			
		HbA1c	0	−	−	−	−	−	−			
		Mixed criteria	6	5206	203	4.28 [2.66–6.24]	3.13 [2.26–4.33]	89.8 [80.5–94.7]	<0.001			0.096
**BMI**												
Median BMI, 23 kg/m^2^	BMI≥23 kg/m^2^	Any criteria	13	5913	408	7.06 [4.66–9.89]	3.72 [3.07–4.50]	92.8 [89.4–95.1]	<0.001	0.002	0.106	0.433
	Biochemical criteria only									0.095		
		OGTT	5	1718	49	2.71 [1.97–3.57]	1.00 [1.00–2.19]	0.0 [0.0–79.2]	0.532		0.175	0.441
		FBG	2	272	39	14.93 [1.49–37.95]	4.36 [2.49–7.62]	94.7 [83.9–98.3]	<0.001		0.314	–
		HbA1c	1	1207	26	2.15 [1.40–3.06]	−	−	−		0.011	–
	Self‐report									0.685		
		Self‐report	2	1206	41	3.38 [2.42–4.49]	1.00	00.0	0.418		0.284	
		Patient folder	1	502	15	2.99 [1.66–4.68]	−	−	−		–	
	Biochemical criteria and/or self‐report									0.418		
		OGTT	2	1054	99	10.97 [2.78–23.55]	5.07 [3.02–8.49]	96.1 [89.1–98.6]	<0.001		0.303	–
		FBG	3	1265	112	8.02 [1.84–17.86]	5.34 [3.70–7.71]	96.5 [92.7–98.3]	<0.001		0.773	0.938
		HbA1c	1	502	35	6.97 [4.90–9.38]	−	−	−		0.439	
		Mixed criteria	2	2276	128	5.60 [4.69–6.59]	1.00 [–]	00.0 [–]	0.909		0.430	
	BMI<23 kg/m^2^	Any criteria	14	13,511	614	4.51 [2.81–6.57]	4.91 [4.20–5.73]	95.8 [94.3–97.0]	<0.001	0.606		0.832
	Biochemical criteria only									<0.001		
		OGTT	1	273	4	1.47 [0.31–3.31]	−	−	−			
		FBG	2	543	36	6.54 [4.57–8.81]	−	−	−			
		HbA1c	1	118	8	6.78 [2.84–12.13]	1.00	00.0	0.498			
		Mixed criteria	1	1316	2	0.15 [0.00–0.46]	−	−	−			
	Self‐report									−		
		Self‐report	3	1678	61	6.43 [1.46–14.36]	3.98 [2.57–6.14]	93.7 [84.9–97.4]	<0.001			
	Biochemical criteria and/or self‐report									<0.001		
		OGTT	2	638	37	5.61 [2.60–9.64]	1.95 [1.00–4.11]	73.7 [0.0–94.1]	0.051			
		FBG	5	5516	397	7.34 [3.96–11.62]	3.76 [2.72–5.21]	92.9 [86.5–96.3]	<0.001			0.978
		RBG	1	1502	7	0.47 [0.17–0.89]	−	−	−			
		HbA1c	1	284	24	8.45 [5.47–11.99]	−	−	−			
		Mixed criteria	5	5577	190	4.53 [2.41–7.26]	4.02 [2.94–5.50]	93.8 [88.5–96.7]	<0.001			0.083
**Area**												
	Combined	Any criteria	21	13,590	765	5.99 [4.68–7.45]	3.23 [2.75–3.80]	90.4 [86.7–93.1]	<0.001	<0.001	0.308	0.163
	Biochemical criteria only									0.126		
		OGTT	4	1612	47	2.81 [2.03–3.70]	1.00 [1.00–2.56]	0.0 [0.0–84.7]	0.395		0.621	0.162
		FBG	3	418	13	3.00 [1.02–5.82]	1.39 [1.00–2.58]	48.4 [0.0–85.0]	0.143		0.002	0.103
		RBG	0	−	−	−	−	−	−			
		HbA1c	1	118	8	6.78 [2.84–12.13]	−	−	−		0.360	
		Mixed criteria	0	−	−	−	−	−	−		0.818	
	Self‐report									0.950		
		Self‐report	7	8212	369	4.36 [3.13–5.78]	2.69 [1.95–3.72]	86.2 [73.7–92.8]	<0.001		<0.001	0.934
		Patient folder	2	832	36	4.46 [1.72–8.35]	2.29 [1.11–4.74]	81.0 [19.0–95.5]	0.021		0.757	
	Biochemical criteria and/or self‐report									0.008		
		OGTT	3	1360	111	8.29 [2.93–15.98]	4.21 [2.76–6.41]	94.4 [86.9–97.6]	<0.001		0.342	0.757
		FBG	7	2824	248	9.48 [6.43–13.04]	2.97 [2.18–4.03]	88.6 [79.0–93.8]	<0.001		0.194	0.098
		HbA1c	2	2328	125	5.72 [3.88–7.87]	1.76 [1.00–3.71]	67.9 [0.0–92.8]	0.077		0.679	
		Mixed criteria	2	1552	69	4.43 [3.46–5.52]	1.00	00.0	0.394		<0.001	
	Urban	Any criteria	37	68,894	2652	4.80 [3.81–5.88]	4.88 [4.44–5.36]	95.8 [94.9–96.5]	<0.001	0.217		0.033
	Biochemical criteria only									0.001		
		OGTT	9	3601	170	3.38 [1.48–5.97]	3.47 [2.72–4.42]	91.7 [86.5–94.9]	<0.001			0.324
		FBG	6	1987	216	10.70 [6.56–15.68]	3.21 [2.33–4.42]	90.3 [81.6–94.9]	<0.001			0.861
		RBG	1	270	33	12.22 [8.56–16.42]	−	−	−			
		HbA1c	5	3537	260	5.04 [1.01–11.78]	7.03 [5.63–8.78]	98.0 [96.8–98.7]	<0.001			0.644
		Mixed criteria	3	5584	272	3.58 [0.00–18.00]	17.99 [15.24–21.22]	99.7 [99.6–99.8]	<0.001			0.624
	Self‐report									0.178		
		Self‐report	5	5419	86	2.64 [0.90–5.21]	4.33 [3.21–5.85]	94.7 [90.3–97.1]	<0.001			0.019
		Patient folder	5	4621	296	5.21 [2.51–8.78]	4.56 [3.41–6.09]	95.2 [91.4–97.3]	<0.001			0.542
	Biochemical criteria and/or self‐report									0.697		
		OGTT	2	572	27	3.47 [0.00–12.74]	4.23 [2.40–7.45]	94.4 [82.6–98.2]	<0.001			
		FBG	6	6074	409	5.53 [3.00–8.74]	3.75 [2.80–5.02]	92.9 [87.2–96.0]	<0.001			0.508
		HbA1c	2	663	28	3.91 [0.00–14.17]	4.81 [2.83–8.18]	95.7 [87.5–98.5]	<0.001			
		Mixed criteria	10	52,459	1585	3.76 [2.62–5.10]	4.53 [3.72–5.51]	95.1 [92.8–96.7]	<0.001			0.526
	Rural	Any criteria	5	3928	142	3.83 [0.94–8.40]	5.59 [4.33–7.23]	96.8 [94.7–98.1]	<0.001	0.382		0.621
	Biochemical criteria only									0.065		
		RBG	1	189	4	2.12 [0.45–4.76]	−	−	−			
		HbA1c	1	500	19	3.80 [2.28–5.67]	−	−	−			
		Mixed criteria	1	1166	21	1.80 [1.11–2.65]	−	−	−			
	Self‐report						−	−	−			
		Self‐report	23	1166	15	1.29 [0.71–2.02]	−	−	−			
	Biochemical criteria and/or self‐report									<0.001		
		FBG	2	1103	45	5.98 [1.02–14.28]	2.56 [1.27–5.18]	84.8 [37.9–96.3]	0.010			
		RBG	1	1502	7	0.47 [0.17–0.89]	−	−	−			
		Mixed criteria	1	1134	86	7.58 [6.11–9.20]	−	−	−			
**Study setting**												
	Clinic‐based	Any criteria	54	77,474	3133	5.21 [4.32–6.19]	4.78 [4.41–5.17]	95.6 [94.9–96.3]	<0.001	0.970	0.193	0.003
	Biochemical criteria only									<0.001		
		OGTT	12	4726	207	3.45 [1.94–5.33]	3.00 [2.39–3.76]	88.9 [82.5–92.9]	<0.001		0.165	0.387
		FBG	9	2405	229	7.89 [4.56–12.00]	3.29 [2.56–4.23]	90.7 [84.7–94.4]	<0.001		−	0.400
		RBG	1	270	33	12.22 [8.56–16.42]					<0.001	
		HbA1c	6	3668	280	5.88 [1.97–11.61]	5.84 [4.67–7.30]	97.1 [95.4–98.1] –	<0.001		0.028	0.748
		Mixed criteria	4	6750	293	3.08 [0.00–12.30]	14.70 [12.57–17.18]	99.5 [99.4–99.7]	<0.001		−	0.541
	Self‐report									0.164		
		Self‐report	8	7669	144	2.93 [1.47–4.85]	4.06 [3.20–5.14]	93.9 [90.2–96.2]	<0.001		0.234	0.001
		Patient folder	7	5453	332	5.00 [2.85–7.71]	3.94 [3.03–5.11]	93.5 [89.1–96.2]	<0.001		−	0.417
	Biochemical criteria and/or self‐report									<0.001		
		OGTT	5	1932	138	6.18 [2.49–11.31]	3.96 [2.89–5.43]	93.6 [88.0–96.6]	<0.001		−	0.846
		FBG	14	10,001	702	7.16 [5.31–9.26]	3.49 [2.88–4.22]	91.8 [87.9–94.4]	<0.001		−	0.683
		RBG	1	1502	7	0.47 [0.17–0.89]	−	−	−		−	
		HbA1c	4	2991	153	4.86 [2.40–8.10]	3.18 [2.11–4.78]	90.1 [77.6–95.6]	<0.001		−	0.998
		Mixed criteria	12	54,011	1654	3.88 [2.84–5.06]	4.21 [3.50–5.07]	94.4 [91.8–96.1]	<0.001		<0.001	0.347
	Community‐based	Any criteria	8	8938	426	4.18 [2.98–5.57]	2.83 [2.12–3.79]	87.6 [77.7–93.1]	<0.001			0.441
	Biochemical criteria only									0.693		
		OGTT	1	487	10	2.05 [0.95–3.53]	−	−	−			
		RBG	1	189	4	2.12 [0.45–4.76]	−	−	−			
		HbA1c	1	487	7	1.44 [0.54–2.72]	−	−	−			
	Self‐report											
		Self‐report	5	7128	326	4.32 [3.10–5.73]	2.54 [1.70–3.79]	84.5 [65.2–93.1]	<0.001			0.761
	Biochemical criteria and/or self‐report											
		Mixed criteria	1	1134	86	7.58 [6.11–9.20]	–	–	–			
**Publication year**												
Median publication year 2018	2018 or later	Any criteria	34	28,020	1547	5.78 [4.42–7.29]	5.00 [4.54–5.51]	96.0 [95.2–96.7]	<0.001	0.559	0.063	0.138
	Biochemical criteria only									0.722		
		OGTT	3	2449	127	3.90 [1.21–7.99]	4.19 [2.75–6.40]	94.3 [86.8–97.6]	<0.001		0.740	0.120
		FBG	6	1863	177	6.72 [3.25–11.27]	3.33 [2.43–4.55]	91.0 [83.1–95.2]	<0.001		0.601	0.067
		HbA1c	5	3480	261	5.56 [1.21–12.65]	7.01 [5.61–8.75]	98.0 [96.8–98.7]	<0.001		0.548	0.765
		Mixed criteria	3	5584	272	3.58 [0.00–18.00]	17.99 [15.24–21.22]	99.7 [99.6–99.8]	<0.001		0.818	0.624
	Self‐report									0.359		
		Self‐report	10	12,925	427	3.64 [2.08–560]	5.13 [4.27–6.15]	96.2 [94.5–97.4]	<0.001		0.686	0.333
		Patient folder	5	2620	166	5.16 [2.60–849]	3.21 [2.25–4.58]	90.3 [80.3–95.2]	<0.001		0.858	0.349
	Biochemical criteria and/or self‐report									<0.001		
		OGTT	5	1932	138	6.18 [2.49–11.31]	3.96 [2.89–5.43]	93.6 [88.0–96.6]	<0.001		−	0.846
		FBG	8	6608	562	10.21 [7.99–12.67]	2.45 [1.79–3.36]	83.3 [68.7–91.1]	<0.001		<0.001	0.029
		RBG	1	1502	7	0.47 [0.17–0.89]	–	–	–			
		HbA1c	4	2991	153	4.86 [2.40–8.10]	3.18 [2.11–4.78]	90.1 [77.6–95.6]	<0.001			0.998
		Mixed criteria	8	6718	195	4.15 [2.05–6.92]	4.60 [3.69–5.73]	94.4 [91.6–96.3]	<0.001		0.737	0.007
	Before 2018	Any criteria	28	58,392	2012	4.18 [3.33–5.12]	3.53 [3.10–4.03]	92.0 [89.6–93.9]	<0.001	0.871		0.055
	Biochemical criteria only									0.012		
		OGTT	10	2764	90	3.12 [1.61–5.06]	2.49 [1.89–3.29]	83.9 [72.0–90.8]	<0.001			0.361
		FBG	3	542	52	10.86 [2.49–23.83]	3.94 [2.55–6.10]	93.6 [84.6–97.3]	<0.001			0.269
		RBG	2	459	37	6.30 [0.14–19.46]	4.33 [2.47–7.58]	94.7 [83.6–98.3]	<0.001			
		HbA1c	2	675	26	3.81 [2.46–5.42]	1.00	00.0	0.833			
		Mixed criteria	1	1166	21	1.80 [0.11–2.65]	–	–	–			
	Self‐report									0.597		
		Self‐report	3	1872	43	2.96 [0.92–6.04]	2.91 [1.75–4.86]	88.2 [67.2–95.8]	<0.001			0.068
		Patient folder	2	2833	166	4.64 [0.63–12.01]	7.13 [4.67–10.89]	98.0 [95.4–99.2]	<0.001			
	Biochemical criteria and/or self‐report									0.566		
		FBG	6	3393	140	3.90 [2.28–5.91]	2.70 [1.90–3.84]	86.3 [72.4–93.2]	<0.001			0.921
		Mixed criteria	5	48,427	1545	4.28 [2.88–5.95]	4.64 [3.48–6.18]	94.8 [91.4–96.8]	<0.001			0.053
**CD4 count level**												
Median CD4 count = 358 cells/μl	CD4 count≥358 cells/μl	Any criteria	14	9805	588	5.55 [3.28–8.36]	4.93 [4.22–5.75]	95.9 [94.4–97.0]	<0.001	0.002	0.298	0.955
	Biochemical criteria only									<0.001		
		OGTT	2	780	25	3.55 [1.34–6.66]	1.46 [1.00–2.93]	53.0 [0.0–88.3]	0.144		0.303	
		FBG	4	843	52	6.05 [4.50–7.80]	1.00 [1.00–2.56]	0.0 [0.0–84.7]	0.520		0.718	0.262
		HbA1c	2	293	15	5.05 [2.65–8.11]	1.05	9.9	0.292		0.111	
		Mixed criteria	1	1166	21	1.80 [1.11–2.65]	−	−	−		<0.001	
	Self‐report									0.309		
		Self‐report	4	2490	73	4.14 [1.55–7.83]	3.61 [2.47–5.29]	92.3 [83.6–96.4]	<0.001		0.164	0.087
		Patient folder	2	884	23	2.58 [1.62–3.75]	1.00	00.0	0.426		0.464	–
	Biochemical criteria and/or self‐report									<0.001		
		OGTT	2	988	49	3.09 [0.01–10.41]	4.13 [2.33–7.33]	94.1 [81.5–98.1]	<0.001		0.218	
		FBG	5	5642	446	9.47 [5.31–14.64]	4.28 [3.17–5.79]	94.5 [90.0–97.0]	<0.001		0.506	0.560
		RBG	1	1502	7	0.47 [0.17–0.89]	−	−	−		–	
		HbA1c	1	502	35	6.97 [4.90–9.38]	−	−	−		0.439	
		Mixed criteria	2	1058	56	5.29 [4.01–6.73]	1.00	00.0			0.704	
	CD4 count<358 cells/μl	Any criteria	13	9974	374	4.06 [2.72–5.65]	3.42 [2.80–4.19]	91.5 [87.2–94.3]	<0.001	0.027		0.289
	Biochemical criteria only									<0.001		
		OGTT	4	1211	28	2.22 [1.43–3.17]	1.00 [1.00–2.56]	0.0 [0.0–84.7]	0.641			0.690
		FBG	2	272	30	10.13 [0.00–44.88]	6.69 [4.31–10.39]	97.8 [94.6–99.1]	<0.001			
		HbA1c	2	1707	45	2.82 [1.42–4.67]	1.88 [1.00–3.96]	71.6 [0.0–93.6]	0.060			
		Mixed criteria	1	1316	2	0.15 [0.00–0.46]	−	−	−			
	Self‐report						−	−	−	0.916		
		Self‐report	1	1316	29	2.20 [1.47–3.07]	−	−	−			
		Patient folder	1	1033	22	2.13 [1.33–3.11]	−	−	−			
	Biochemical criteria and/or self‐report						−	−	−	0.229		
		OGTT	1	332	25	7.53 [4.91–10.64]	−	−	−			
		FBG	2	740	58	7.78 [5.78–10.05]	1.09	16.5	0.273			
		HbA1c	1	284	24	8.45 [5.47–11.99]	−	−	−			
		Mixed criteria	5	7050	277	4.78 [2.65–7.47]	4.58 [3.43–6.12]	95.2 [91.5–97.3]	<0.001			0.167
**ART use**												
	Combined	Any criteria	18	18,294	926	5.38 [3.82–7.18]	4.92 [4.29–5.64]	95.9 [94.6–96.9]	<0.001	0.928	0.827	0.142
	Biochemical criteria only									<0.001		
		OGTT	2	967	23	2.18 [1.02–3.75]	1.36	46.3	0.172		0.404	
		FBG	2	1020	134	12.97 [8.38–18.37]	2.42 [1.18–4.95]	82.9 [28.8–95.9]	0.015		0.006	
		RBG	1	189	4	2.12 [0.45–4.76]					<0.001	
		HbA1c	1	500	19	3.80 [2.28–5.67]	−	−	−		0.644	
		Mixed criteria	1	2979	13	0.44 [0.23–0.71]	−	−	−		<0.001	
	Self‐report									0.001		
		Self‐report	9	11,739	396	3.43 [1.84–5.48]	5.21 [4.31–6.31]	96.3 [94.6–97.5]	<0.001		0.918	0.484
		Patient folder	2	2130	165	7.70 [6.60–8.88]	1.00	00.0	0.324		0.005	
	Biochemical criteria and/or self‐report											
		OGTT	1	748	47	6.28 [4.65–8.14]	−	−	−		0.956	
		FBG	3	1719	96	6.60 [2.87–11.67]	3.43 [2.14–5.50]	91.5 [78.2–96.7]	<0.001		0.847	0.196
		Mixed criteria	3	5065	150	3.61 [0.50–9.31]	7.95 [5.97–10.59]	98.4 [97.2–99.1]	<0.001		0.304	0.283
	No ART	Any criteria	17	48,048	1633	5.16 [3.56–7.01]	4.05 [3.46–4.74]	93.9 [91.6–95.6]	<0.001	0.927		0.040
	Biochemical criteria only									<0.001		
		OGTT	5	2220	119	3.22 [0.96–6.67]	3.60 [2.58–5.02]	92.3 [85.0–96.0]	<0.001			0.015
		FBG	2	244	33	12.67 [0.00–41.12]	5.21 [3.14–8.66]	96.3 [89.8–98.7]	<0.001			
		HbA1c	3	2476	200	5.53 [0.04–18.54]	9.93 [7.75–12.73]	99.0 [98.3–99.4]	<0.001			0.901
		Mixed criteria	1	954	223	23.38 [20.74–26.12]	−	−	−			
	Self‐report						−	−	−			
		Self‐report	0	−	−	−	−	−	−			
		Patient folder	1	244	8	3.28 [1.65–6.42]	−	−	−			
	Biochemical criteria and/or self‐report									<0.001		
		OGTT	2	638	37	5.61 [2.60–9.64]	1.95 [1.00–4.11]	73.7 [0.0–94.1]	0.051			
		FBG	5	1112	98	8.07 [4.09–13.17]	2.65 [1.79–3.93]	85.8 [68.8–93.5]	<0.001			0.964
		RBG	1	285	1	0.35 [0.00–1.50]	−	−	−		0.960	
		HbA1c	2	528	40	7.55 [5.42–9.98]	1.00	00.0	0.421		0.100	
		Mixed criteria	4	44,213	1411	6.03 [3.12–9.77]	5.25 [3.87–7.13]	96.4 [93.3–98.0]	<0.001			0.138
	ART use	Any criteria	35	20,070	1000	4.72 [3.54–6.05]	3.99 [3.57–4.45]	93.7 [92.2–94.9]	<0.001	0.761		0.652
	Biochemical criteria only									0.001		
		OGTT	8	2026	75	3.79 [1.78–6.45]	2.63 [1.94–3.56]	85.5 [73.4–92.1]	<0.001			0.221
		FBG	5	1141	62	4.83 [2.83–7.30]	1.68 [1.04–2.72]	64.5 [6.7–86.5]	0.023			0.415
		RBG	1	270	33	12.22 [8.56–16.42]	−	−	−			
		HbA1c	5	1179	68	5.03 [2.79–7.84]	1.91 [1.20–3.02]	72.5 [30.9–89.0]	0.005			0.385
		Mixed criteria	3	2817	57	2.67 [0.06–8.50]	6.67 [4.84–9.19]	97.8 [95.7–98.8]	<0.001			0.265
	Self‐report											
		Self‐report	4	3058	74	3.52 [1.43–6.45]	3.40 [2.29–5.03]	91.3 [80.9–96.1]	<0.001			0.089
		Patient folder	5	3079	159	4.17 [1.56–7.90]	4.13 [3.04–5.62]	94.1 [89.2–96.8]	<0.001			0.694
	Biochemical criteria and/or self‐report									<0.001		
		OGTT	2	546	54	6.66 [0.00–30.20]	7.54 [5.01–11.36]	98.2 [96.0–99.2]	<0.001			
		FBG	10	7170	508	6.90 [4.63–9.58]	3.41 [2.71–4.31]	91.4 [86.3–94.6]	<0.001			0.979
		RBG	1	1217	6	0.49 [0.16–0.98]	−	−	−			
		HbA1c	3	2463	113	3.96 [1.32–7.87]	3.30 [2.04–5.34]	90.8 [76.0–96.5]	<0.001			0.831
		Mixed criteria	7	5867	179	3.48 [2.42–4.72]	2.11 [1.46–3.05]	77.6 [53.4–89.2]	<0.001			0.102
**ART duration**												
Median duration of ART use = 4.5 years	On ART for ≥4.5 years	Any criteria	12	9824	514	4.56 [2.77–6.74]	4.21 [3.49–5.07]	94.4 [91.8–96.1]	<0.001	0.031	0.550	0.570
	Biochemical criteria only									<0.001		
		OGTT	1	150	27	18.00 [12.23–24.59]					<0.001	
		FBG	4	977	51	4.41 [2.16–7.35]	1.87 [1.11–3.15]	71.3 [18.1–89.9]	0.015		−	0.355
		RBG	1	270	33	12.22 [8.56–16.42]	−	−	−		−	
		HbA1c	2	497	33	6.57 [4.52–8.96]	1.00	00.0	0.872		−	
		Mixed criteria	1	1316	1	0.15 [0.00–0.46]	−	−	−		0.001	
	Self‐report								0.096			
		Self‐report	3	1856	63	5.48 [1.63–11.29]	3.69 [2.35–5.81]	92.7 [81.9–97.0]‐	<0.001		0.101	0.102
		Patient folder	2	1415	30	2.10 [1.40–2.93]	1.00	00.0	0.962		0.280	
	Biochemical criteria and/or self‐report									0.005		
		OGTT	1	240	2	0.83 [0.01–2.50]	−	−	−		<0.001	
		FBG	4	5844	379	6.35 [2.96–10.87]	4.64 [3.34–6.45]	95.4 [91.0–97.6]	<0.001		0.960	0.839
		HbA1c	1	379	4	1.06 [0.22–2.39]	−	−	−		<0.001	
		Mixed criteria	2	1916	61	3.50 [1.36–6.56]	2.92 [1.49–5.72]	88.3 [55.2–96.9]	0.003		0.255	
	On ART for <4.5 years	Any criteria	12	7191	300	3.68 [1.89–5.99]	4.37 [3.65–5.24]	94.8 [92.5–96.4]	<0.001	0.234		0.725
	Biochemical criteria only									0.336		
		OGTT	4	926	22	2.28 [1.14–3.75]	1.21 [1.00–2.02]	31.7 [0.0–75.5]	0.222			0.450
		Mixed criteria	1	1166	21	1.80 [1.11–2.65]	−	−	−			
	Self‐report									0.325		
		Self‐report	2	1624	28	1.90 [0.66–3.74]	2.06 [1.00–4.32]	76.5 [0.0–94.6]	0.039			
		Patient folder	1	502	15	2.99 [1.66–4.68]	−	−	−			
	Biochemical criteria and/or self‐report									<0.001		
		OGTT	1	332	25	7.53 [4.91–10.64]	−	−	−			
		FBG	3	1234	95	6.16 [0.85–15.63]	5.50 [3.84–7.89]	96.7 [93.2–98.4]	<0.001			0.633
		RBG	1	1502	7	0.47 [0.17–0.89]	−	−	−			
		HbA1c	2	786	59	7.48 [5.73–9.44]	1.00	00.0	0.439			
		Mixed criteria	5	3861	217	5.61 [3.38–8.35]	2.31 [1.70–3.14]	81.3 [65.6–89.9]	<0.001			0.786

Abbreviations: FBG, fasting blood glucose; OGTT, oral glucose tolerant test; RBG, random blood glucose.

– not computable.

Two overall pooled prediabetes prevalence estimates were calculated across studies that utilized HbA1c (*n* = 30) and those that did not (*n* = 24). The six studies that diagnosed prediabetes using HbA1c alone were excluded from the sub‐group analyses [31, 38, 51, 59, 62, 67] (Tables S2 and S3). Among the studies that described prediabetes, IGT was the most frequently reported form, followed by IFG.

### 3.5 Prevalence of diabetes

**Figure 3:**
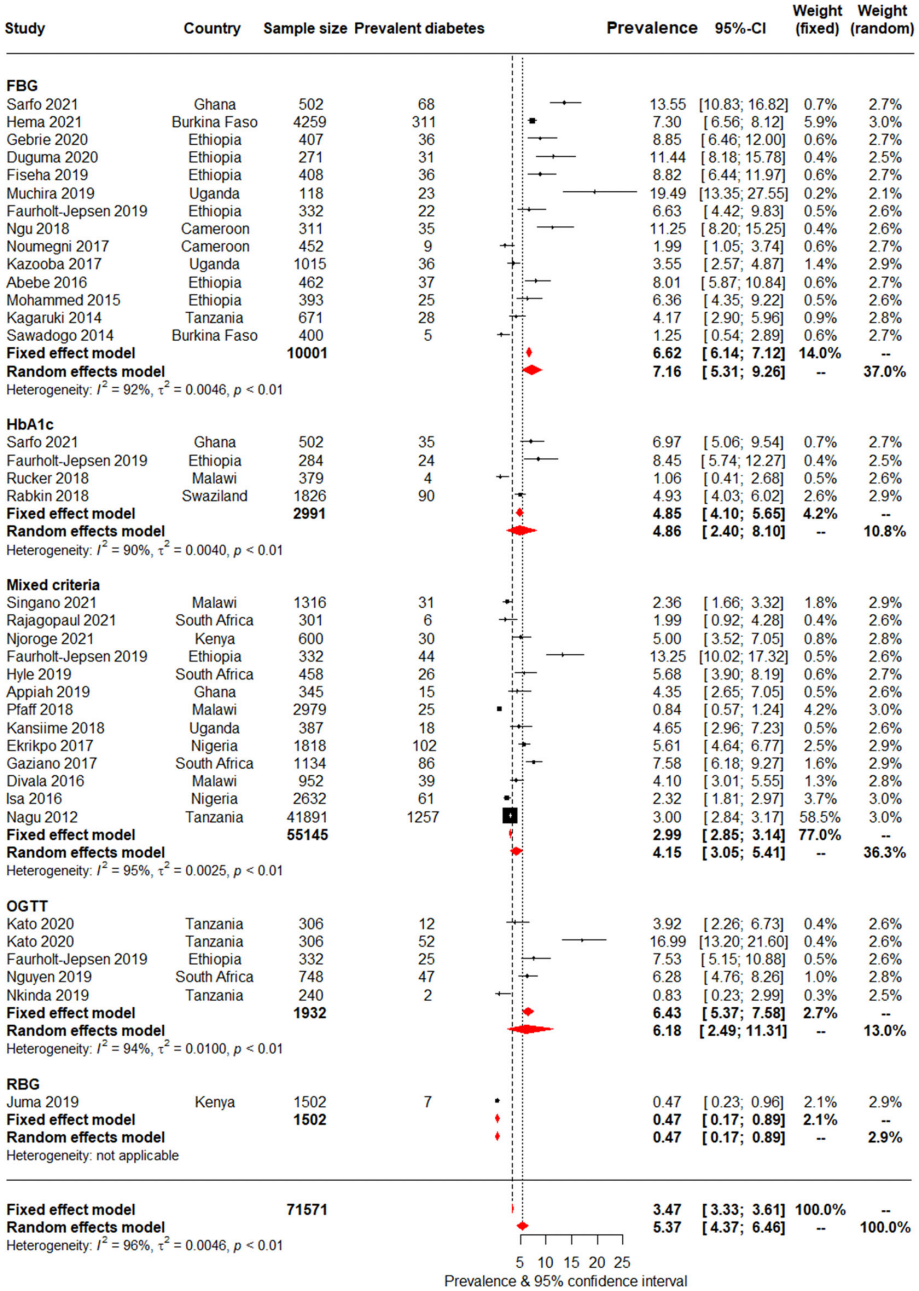
Pooled prevalence of diabetes across studies using biochemical tests and/or self‐reports to diagnose diabetes. Each diagnostic criterion included biochemical test and/or self‐reported diabetes. For each study, the black box represents the study estimate (prevalence of diabetes) and the horizontal bar denotes the 95% confidence intervals (95% CI). The size of the boxes is proportional to the inverse variance. The diamonds at the lower tail of the figure are for the pooled effect estimates from both random and fixed effects models. The proportional contribution of each study (weight) to the pooled estimates is also shown separately for fixed and random effects models, together with the prevalence estimates and measures of heterogeneity. The vertical line is centred on the pooled estimates.

The diabetes prevalence rates by biochemical tests and/or self‐reported diabetes are illustrated in the forest plots in Figure 3. Overall, 3559 of the 86,412 participants included in the overall pooled estimate had diabetes, corresponding to a prevalence of 5.1% (95% CI: 4.3–5.9). Self‐reported diabetes prevalence per se, at 3.5% (2.2–5.1), was much lower than when combined with biochemical assessments (OGTT and/or self‐report: 6.2% [2.5–11.3]; FBG and/or self‐report: 7.2% [5.3–9.3]). For these data, the *I* ^2^ was between 92% and 95%, and the *p*‐*heterogeneity* was <0.001.

Although not significantly different, diabetes prevalence was generally higher in participants who were older (cut‐point 39 years: 6.0% [4.5–7.6] vs. 4.5% [3.5–5.7]), had higher BMI (cut‐point 23 kg/m^2^: 7.1% [4.7–9.9] vs. 4.5% [2.8–6.6]), lived in urban versus rural areas (4.8% [3.8–5.9] vs. 3.8% [0.9–8.4]) and in studies published after versus before 2018 (5.8% [4.4–7.3] vs. 4.2% [3.3–5.1]). Diabetes prevalence was also not significantly different by HIV‐related factors of CD4 count, ART use or duration of ART use. There was also substantial heterogeneity for the diabetes prevalence by sub‐group analyses; the *I* ^2^ was between 92% and 97%, and *p*‐*heterogeneity* <0.001.

For the overall diabetes prevalence, there was some evidence of publication bias overall (*p* = 0.002 for the Egger test). There was also evidence of bias among studies conducted in clinic‐based settings (*p* = 0.003), urban areas (*p* = 0.033) and in participants younger than 40 years of age (*p* = 0.002). In trim and fill analyses however, imputed studies always had implausible effect estimates, with diabetes prevalence always lower than 1%, and null in about half of imputed studies (Figures S1–S4). This is unlikely and suggests that publication bias was a spurious finding (Figure S5).

### 3.6 Prevalence of prediabetes

**Figure 4:**
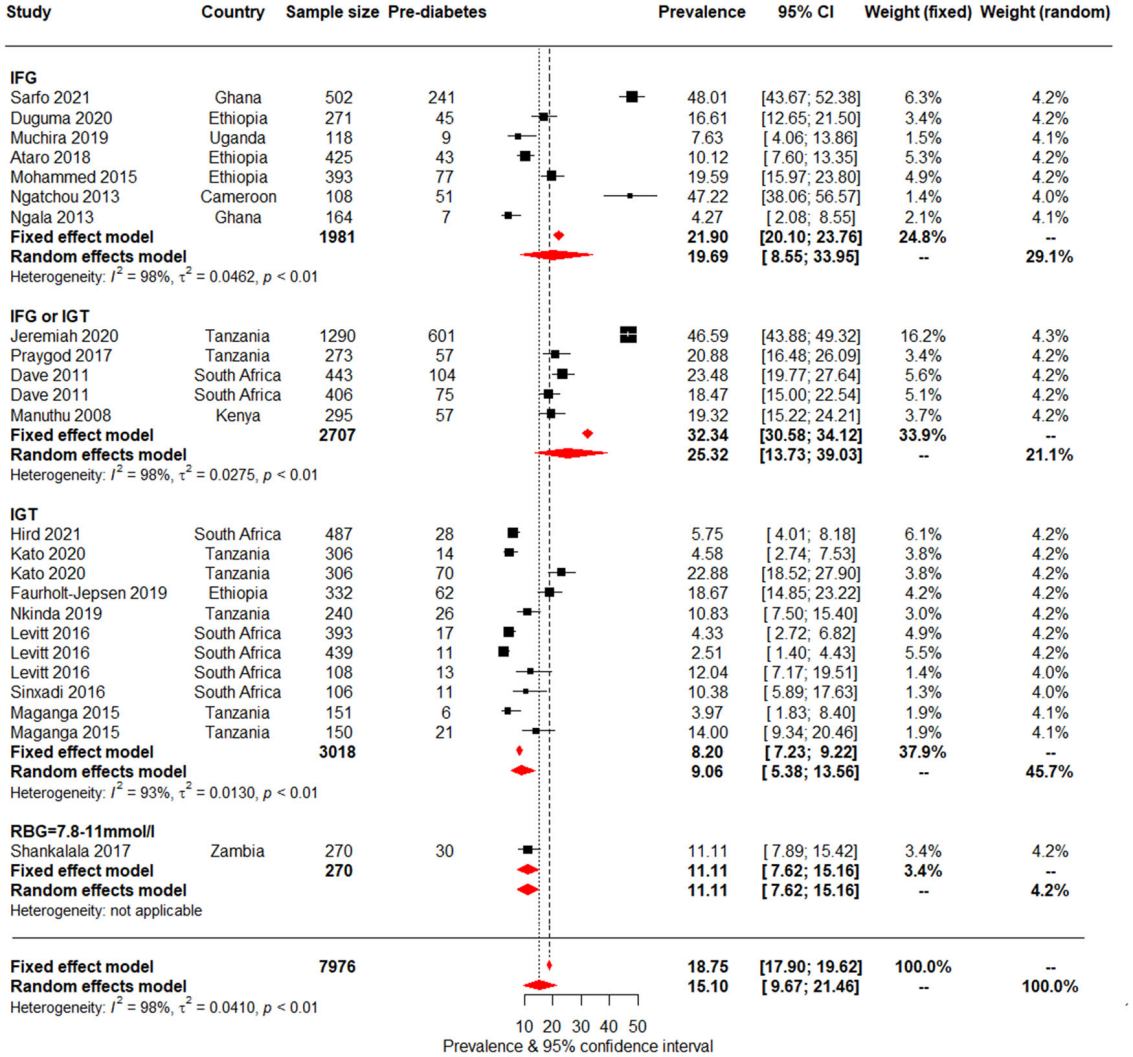
Pooled prediabetes prevalence in people living with HIV, presented by biochemical tests. For each study, the black box represents the study estimate (prevalence of diabetes) and the horizontal bar denotes the 95% confidence intervals (95% CI). The size of the boxes is proportional to the inverse variance. The diamonds at the lower tail of the figure are for the pooled effect estimates from both random and fixed effects models. The proportional contribution of each study (weight) to the pooled estimates is also shown separately for fixed and random effects models, together with the prevalence estimates and measures of heterogeneity. The vertical line is centred on the pooled estimates.

The prediabetes prevalence is presented in Table S4 and Figure 4. Prevalence was similarly high across studies that did not use HbA1c and those that did: 15.1% (95% CI: 9.7–21.5) versus 15.2% (10.8–20.1). There was no significant difference in prediabetes prevalence by sub‐groups (Table S4). However, prediabetes was higher in older (≥39 years) compared with younger participants (22.5% [11.6–35.7] vs. 9.7% [5.5–14.8]), in women compared with men (10.0% [3.9–18.5] vs. 6.2% [0.3–17.6]), in those with higher BMI (≥25 kg/m^2^) compared with BMI <25 kg/m^2^ (18.1% [5.2–36.2] vs. 10.3% [3.9–18.5]) and in publications after 2017 than before (17.1% [8.7–27.5] vs. 13.2% [7.7–19.8]). Prediabetes prevalence was similar by CD4 counts, ART use and duration of ART use, although point‐estimates were higher in ART naïve and participants with shorter duration of ART use.

Considerable heterogeneity was also apparent across studies for prediabetes prevalence with *I* ^2^ between 95% and 99%, and *p*‐*heterogeneity* <0.001. There was some evidence of publication bias when studies that used HbA1c alone were not accounted for (*p* = 0.015 for the Egger test), but not when these studies were included in the analysis (*p* = 0.197). Evidence of bias was also apparent across clinic‐based studies (*p* = 0.008) and those in urban settings (*p* = 0.017). In trim and fill analyses, imputed studies systematically had very high effect estimates, with prediabetes prevalence of 50% or higher. This is implausible and suggests that findings of publication bias were spurious findings (Figures S6–S8).

## 4 DISCUSSION

This systematic review and meta‐analysis conducted in adult PLHIV in Africa illustrates an established burden of diabetes, at 5.1%, and a high prediabetes prevalence of 15.2%. This diabetes prevalence accords with the 5.3% age‐adjusted diabetes prevalence in the Africa region reported in the 2021 International Diabetes Federation (IDF) Atlas [70]. The age‐adjusted IGT and IFG prevalence rates in the Africa region were 12.6% and 8.0%, respectively. The comparative prevalence rates of diabetes and prediabetes in PLHIV compared with general populations likely suggest similar influences in their development of dysglycaemia.

The prevalence of self‐reported diabetes only (3.5%), which reflects diabetes awareness or detection rather than true prevalence, was lower than combined self‐report with biochemically assessed diabetes (OGTT: 6.2%; FBG: 7.2%). Further, although not statistically significant, general trends in sub‐group analyses conformed with traditional diabetes risk factors and were in the expected direction; prevalence rates were higher with older age, greater BMI and in urban residents. Notably, a rising prevalence of diabetes and prediabetes over time was suggested by higher rates in recent versus earlier publications. There were no clear trends for diabetes and prediabetes prevalence by HIV‐related factors.

The lower prevalence of known or self‐reported diabetes compared with diabetes prevalence identified on combined biochemical analyses with self‐report suggests that a substantial proportion of PLHIV with co‐morbid diabetes were undiagnosed for the latter condition. This is likely similar to general populations in Africa where a substantial proportion of diabetes is undiagnosed [9, 10]. However, unlike general populations, these PLHIV are in regular contact with health services and would be expected to have all co‐morbidities, including diabetes identified. Unfortunately, in practice, ART is generally provided by international donors in Africa with little funding or care provided for NCDs [71]. Consequently, there are disparities in management with the free treatment provided for HIV but a minimal focus on diabetes and other CVDs, such as hypertension and dyslipidaemia.

This is a missed opportunity to holistically manage the rise in NCD comorbidities in PLHIV in Africa. Policymakers should be alerted to the tangible shift in approach that is urgently required for the care of this vulnerable population. There needs to be a swing from a focus on HIV itself to a more comprehensive approach that encompasses the care of neglected NCD co‐morbidities. This is important if the momentum gained in increasing life expectancy in PLHIV in Africa is to be maintained. Screening for diabetes should be included in routine assessments of PLHIV in Africa, which is currently not standard practice [8].

The urgent need for this shift in approach for the care of diabetes and other NCD co‐morbidities in African PLHIV is underscored by the high burden of prediabetes demonstrated in this review; almost one in six people were affected. Generally, it is predicted that 5–10% of individuals with prediabetes will progress to diabetes annually [72]. This likely foretells of a substantial increase in diabetes prevalence in this vulnerable population in future. The effectiveness of HAART with increased longevity and subsequent ageing, and the uptake of unhealthy lifestyle behaviours will likely translate to the high prediabetes burden progressing to diabetes. A recent systematic review demonstrated that, similar to general populations, traditional risk factors, such as older age, diabetes family history, overweight/obesity and so on, were among the main contributors to the development of dysglycaemia in PLHIV globally, including in Africa [5]. The higher prevalence of diabetes and prediabetes with older age and higher BMI in the current review likely corroborates the influence of traditional risk factors in the development of dysglycaemia in African PLHIV. Therefore, the large burden of prediabetes in African PLHIV with the potential for conversion to diabetes in the future possibly mirrors the diabetes trends predicted for general populations in Africa.

Reinforcing the future expansion of diabetes in PLHIV in Africa, although not significant, was the higher prevalence of diabetes and prediabetes illustrated in recent years. This increasing pattern is likely a reflection of the diabetes trend predicted in general populations in Africa. The 4.7% diabetes prevalence estimated in Africa in 2019 is expected to rise to 5.2% by 2045 with a more than doubling of the absolute numbers [10]. The current literature and this review likely underline a shift in the disease burden from communicable diseases to NCDs in Africa with diabetes a significant disease entity in the region [9, 73], even in PLHIV.

Similar to the findings of a systematic review conducted a few years ago but using different eligibility criteria for included studies [4], the current review found no statistically significant difference in diabetes prevalence by ART status. Two additional systematic reviews, one conducted in Sub‐Saharan Africa [6] and the other in a few longitudinal studies in PLHIV globally [8], reported no association between ART use and FBG. Nevertheless, the uncertainty of the evidence is highlighted by the overall findings in the review by Nduka and colleagues, which included mainly cross‐sectional studies. They reported an association between ART use and diabetes, diagnosed on mean FBG levels [8]. Moreover, a systematic review by Nansseu and colleagues of longitudinal studies conducted in PLHIV globally reported an association between a cumulative exposure to some ART drugs and incident diabetes and prediabetes, but this finding was not consistent across studies [5]. Despite their differences in the eligibility criteria for included studies, these systematic reviews underscore the absence of clear irrefutable evidence linking the development of diabetes with ART [74].

Over the last few years, there has been a change to the use of newer ART drugs with fewer metabolic effects [5]. Studies conducted in populations using newer drugs would have been unlikely to be included in the reviews of studies published prior to 2017. Further research detailing the newer ART drugs used in recent studies and their specific contributions to the development of diabetes, if any, is required. This includes dolutegravir, an integrase inhibitor, which has been found to be more effective and better tolerated than older ART medications, leading to its recommended use as a preferred first‐ and second‐line ART by the World Health Organization [75]. Recent evidence from Africa describes greater odds of hyperglycaemia in PLHIV treated with dolutegravir compared with other ART regimens even after adjusting for potential confounders of age, BMI and co‐morbid hypertension [75]. If a wider body of research confirms these findings, systematic screening for diabetes and prediabetes prior to the use of dolutegravir may need to be incorporated into HIV treatment guidelines [75].

### 4.1 Strengths and limitations

The strengths of this review include the following: (1) using a review protocol with a comprehensive and systematic search strategy examining five separate databases and the reference lists of eligible studies; (2) evaluating a large number of participants from different studies; and (3) using the Freeman–Tukey double arc‐sine transformation which stabilized the variance of primary studies before combining the data; this limited the effect on the pooled estimates of studies with small or large prevalence rates.

The limitations of this review include the following: (1) the restriction to English and French languages may have excluded eligible studies in other languages and introduced a language bias; (2) the inability to examine, because of insufficient data, the associations by ART drug category, which may have been clinically relevant; (3) the inability to describe, because of insufficient data, the associations by adiposity category, which may have underscored the importance of the relation of traditional risk factors with diabetes; (4) the inclusion of only cross‐sectional studies precluded any causal inferences; (5) few (six) eligible studies had a low risk of bias; (6) the substantial heterogeneity among included studies; and (7) the inability to explore the association with a family history of diabetes, which is a key risk factor for diabetes; this was because of insufficient data.

## 5 CONCLUSIONS

As the diabetes epidemic worsens in Africa, adult PLHIV are affected as severely, and by similar socio‐demographic and anthropometric factors, as Africans without HIV. Furthermore, the high prevalence of prediabetes portends a likely increase in future diabetes. Policymakers in African countries must be alerted to the need to integrate cost‐effective and efficient screening, prevention and treatment of diabetes with HIV care; this will maintain the momentum and secure the advances made in optimizing HIV management. Otherwise, the future will witness a substantial proportion of PLHIV in Africa succumbing to premature diabetes and CVD‐related morbidity and mortality. Evidence‐based research is needed to provide guidance on the best strategies and approaches for the integration of diabetes and CVD prevention and care with HIV management. This review, comprising cross‐sectional studies, highlights the lack of associations between diabetes and HIV‐related factors of CD4 count, ART use and duration of ART use. Longitudinal studies are, therefore, needed to clearly elucidate the influences, both traditional and HIV related, on the development of diabetes in African PLHIV.

## COMPETING INTERESTS

None to declare for all co‐authors.

## AUTHORS’ CONTRIBUTIONS

Study conception (NP, KAN and APK), protocol drafting (KAN, NP and APK), protocol operationalization (NP, KAN and APK), data analysis and interpretation (KAN, NP and APK), drafting the manuscript (NP, KAN and APK), critical revision of the manuscript (JH, AES, JCC and JBN) and approval of the final version (all co‐authors).

## FUNDING

NP, KAN, JH and APK are supported by the South African Medical Research Council. AES is supported by the intramural programs of the National Institute of Diabetes and Digestive and Kidney Diseases and the National Institute of Minority Health and Health Disparities of the National Institutes of Health (NIH, Bethesda, Maryland, USA).

## DATA ACCESS, RESPONSIBILITY AND ANALYSIS

KAN and APK had full access to all the data in the study and take responsibility for the integrity of the data and accuracy of the data analysis; both are guarantors.

## Supporting information


**Figure S1**: Forest plot showing the overall pooled prevalence of diabetes in people living with HIV, from the trim and fill analyses.

**Figure S2**: Forest plot showing the pooled prevalence of diabetes in people living with HIV in clinic‐based studies, from the trim and fill analyses.

**Figure S3**: Forest plot showing the pooled prevalence of diabetes in people living with HIV in urban settings, from the trim and fill analyses.

**Figure S4**: Forest plot showing the pooled prevalence of diabetes in people younger than 40 years old living with HIV, from the trim and fill analyses .

**Figure S5**: Funnel plots for studies that reported prevalence of diabetes in people living with HIV (A) overall and (B) in clinic‐based settings from the trim and fill analyses. Black dots identify the actual studies while clear dots identify imputed studies.

**Figure S6**: Forest plot showing the pooled prevalence of pre‐diabetes in people living with HIV, from the trim and fill analyses.

**Figure S7**: Forest plot showing the pooled prevalence of pre‐diabetes in people living with HIV in studies in clinical settings, from the trim and fill analyses.

**Figure S8**: Forest plot showing the pooled prevalence of pre‐diabetes in people living with HIV in studies in urban areas, from the trim and fill analyses.

## Data Availability

The study is based on aggregation of publicly available data from primary studies. As such, there are no data to be shared.
