# Supplementary figures and images for: Prevalence and influences of diabetes and prediabetes among adults living with HIV in Africa: a systematic review and meta‐analysis
Source: J Int AIDS Soc. 2023 Mar 16;26(3):e26059. doi: 10.1002/jia2.26059 (PMC10018386; doi:10.1002/jia2.26059)

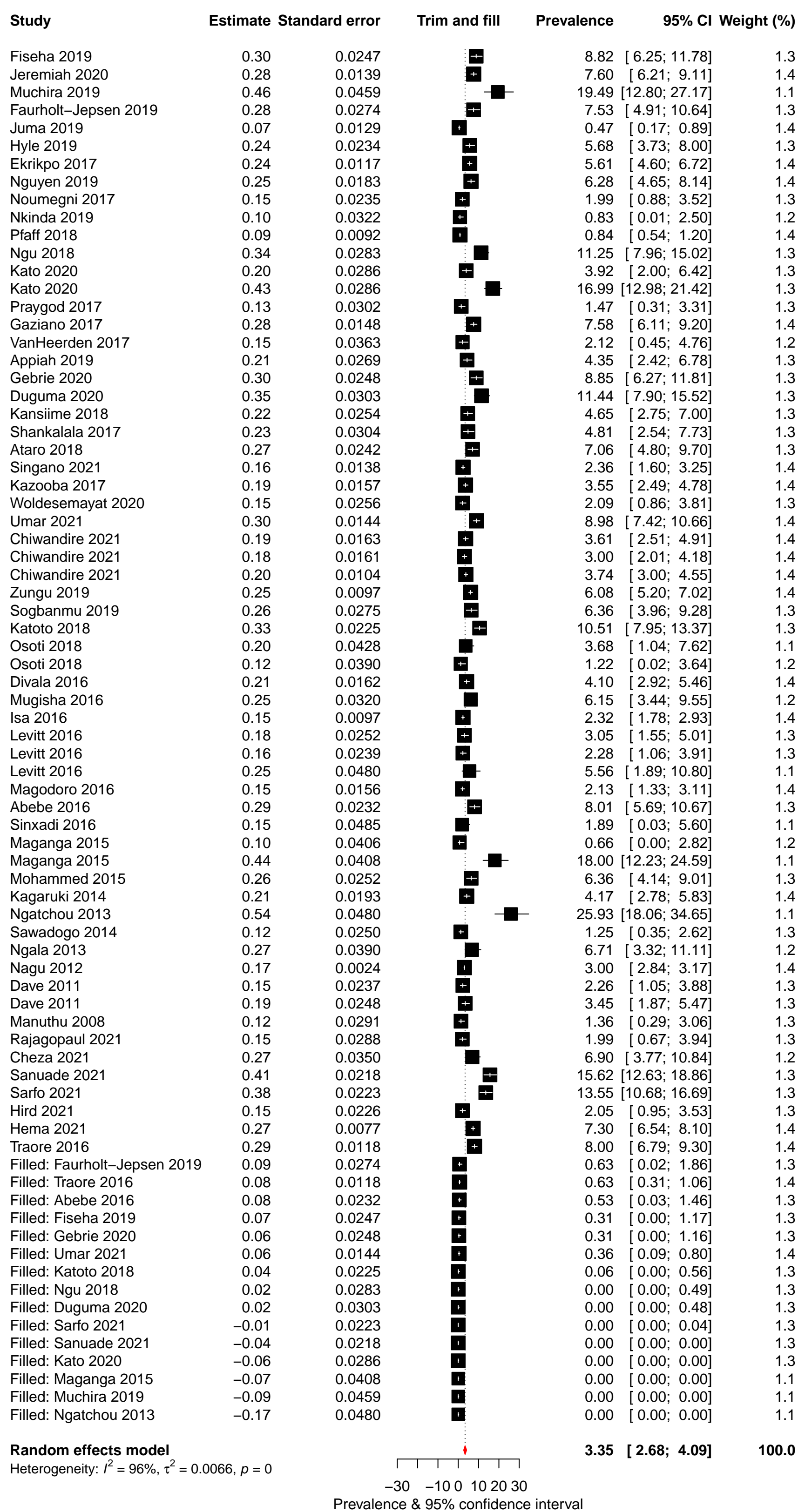

Supplement: Supplementary file 1 — Figure S1: Forest plot showing the overall pooled prevalence of diabetes in people living with HIV, from the trim and fill analyses. [file JIA2-26-e26059-s006.pdf]

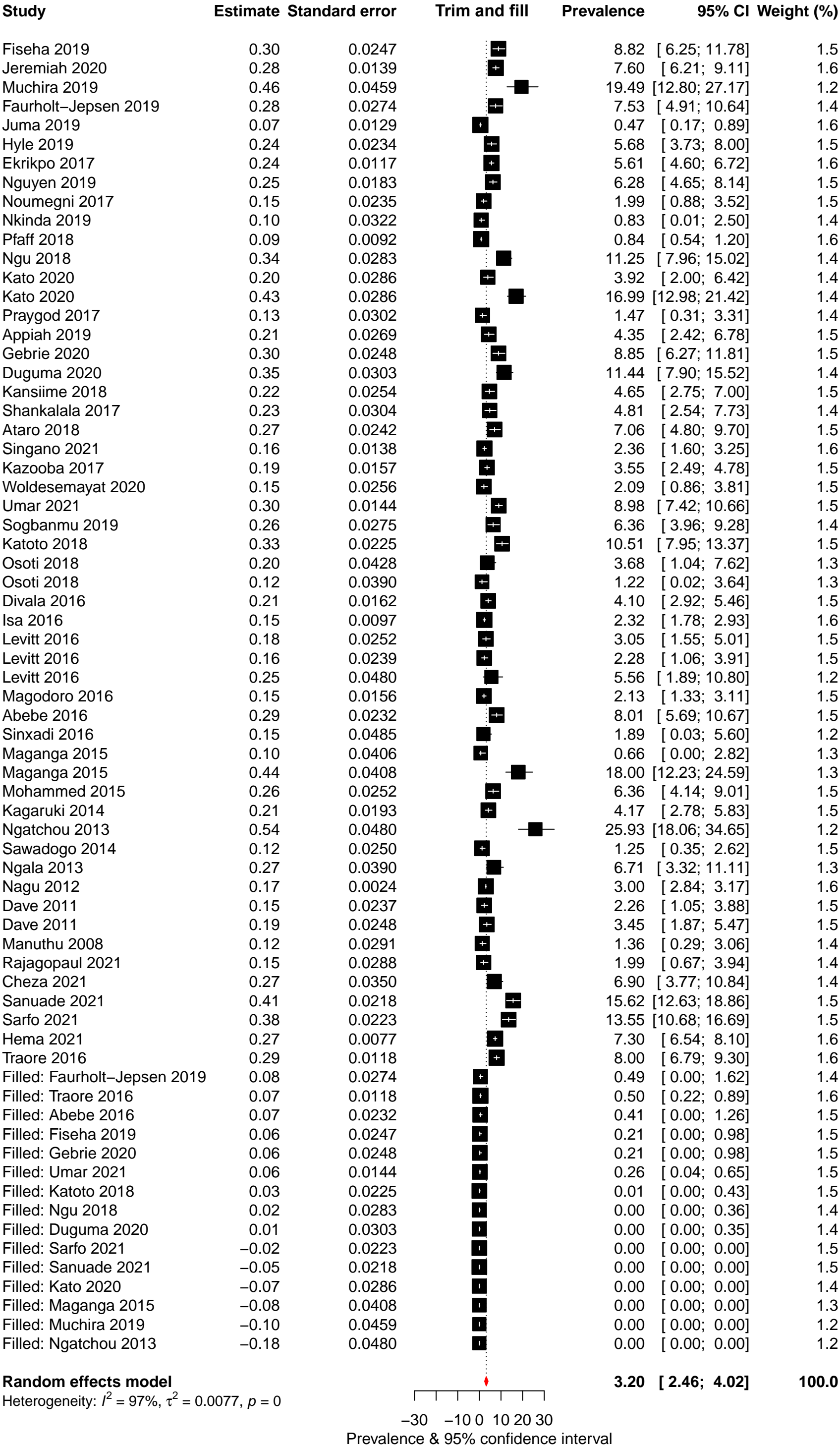

Prevalence & 95% confidence interval

Supplement: Supplementary file 2 — Figure S2: Forest plot showing the pooled prevalence of diabetes in people living with HIV in clinic‐based studies, from the trim and fill analyses. [file JIA2-26-e26059-s008.pdf]

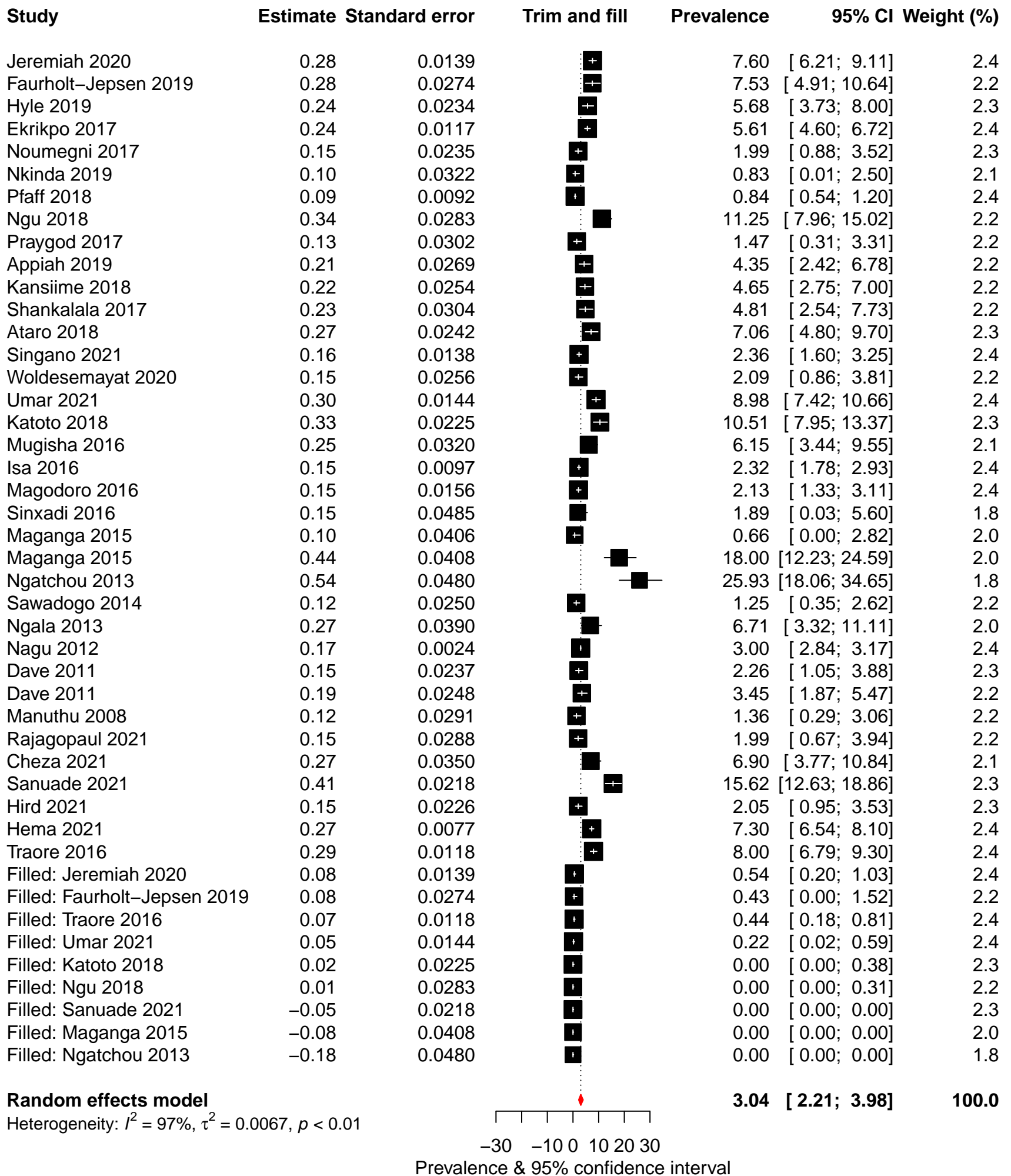

Supplement: Supplementary file 3 — Figure S3: Forest plot showing the pooled prevalence of diabetes in people living with HIV in urban settings, from the trim and fill analyses. [file JIA2-26-e26059-s003.pdf]

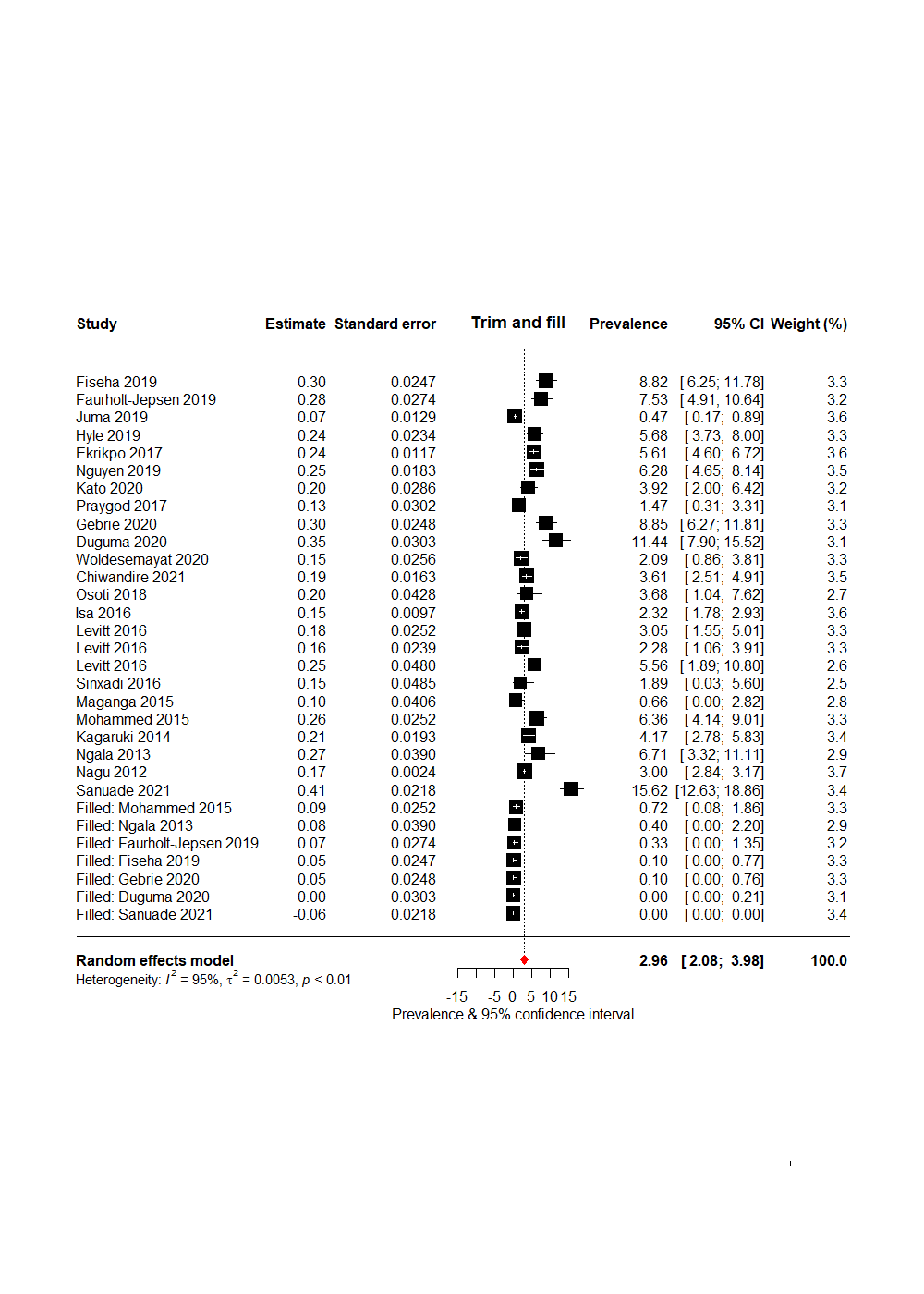

Supplement: Supplementary file 4 — Figure S4: Forest plot showing the pooled prevalence of diabetes in people younger than 40 years old living with HIV, from the trim and fill analyses . [file JIA2-26-e26059-s007.tif]

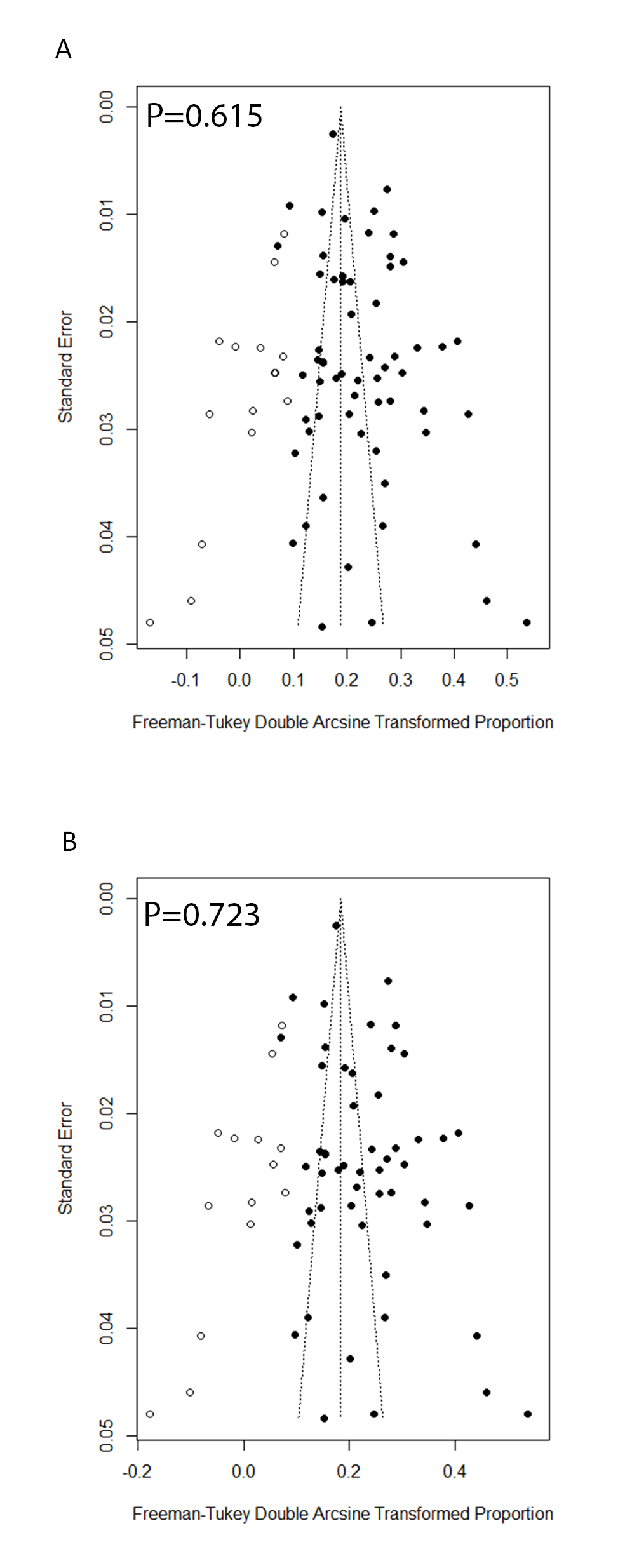

Supplement: Supplementary file 5 — Figure S5: Funnel plots for studies that reported prevalence of diabetes in people living with HIV (A) overall and (B) in clinic‐based settings from the trim and fill analyses. Black dots identify the actual studies while clear dots identify imputed studies. [file JIA2-26-e26059-s002.png]

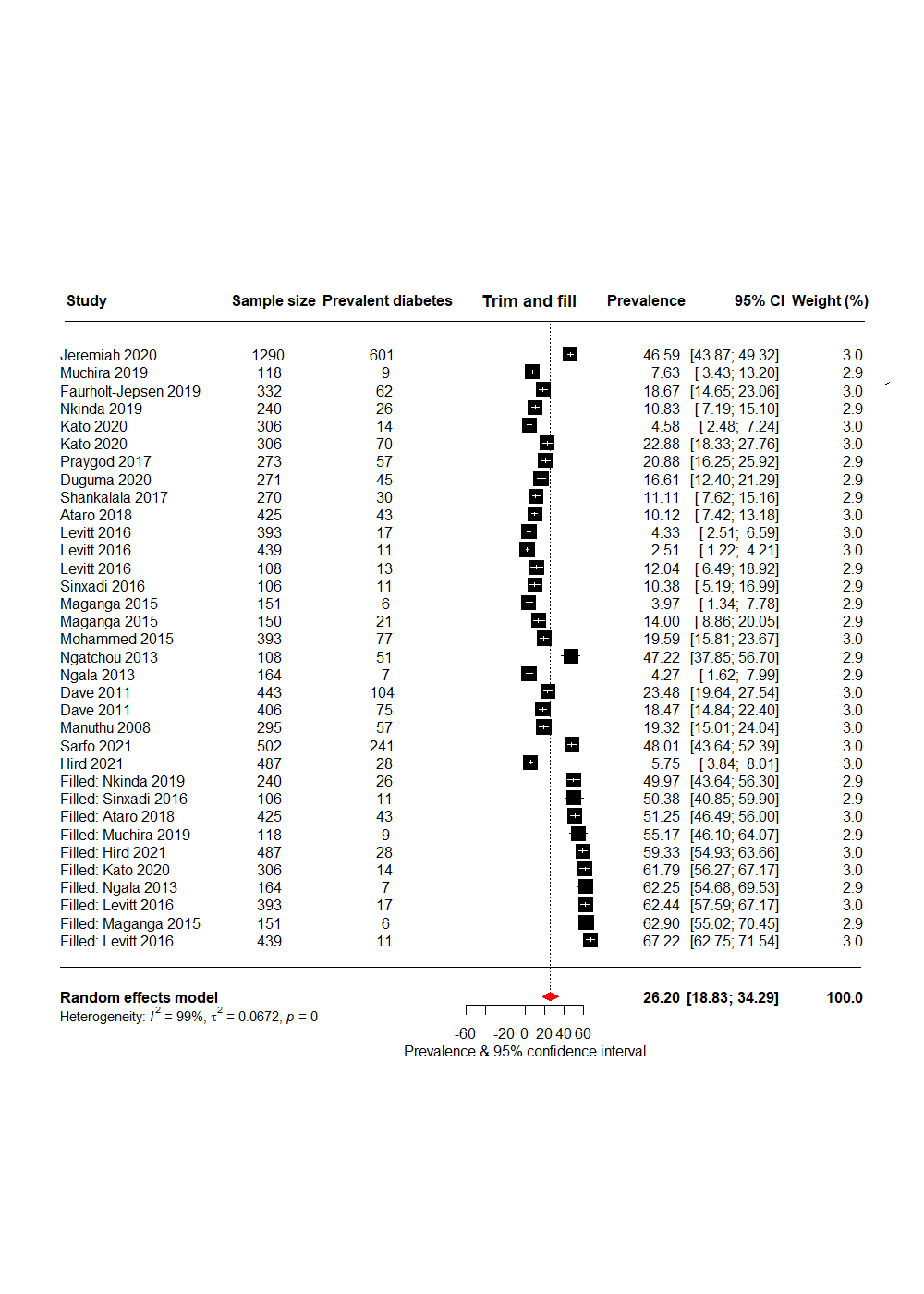

Supplement: Supplementary file 6 — Figure S6: Forest plot showing the pooled prevalence of pre‐diabetes in people living with HIV, from the trim and fill analyses. [file JIA2-26-e26059-s001.tif]

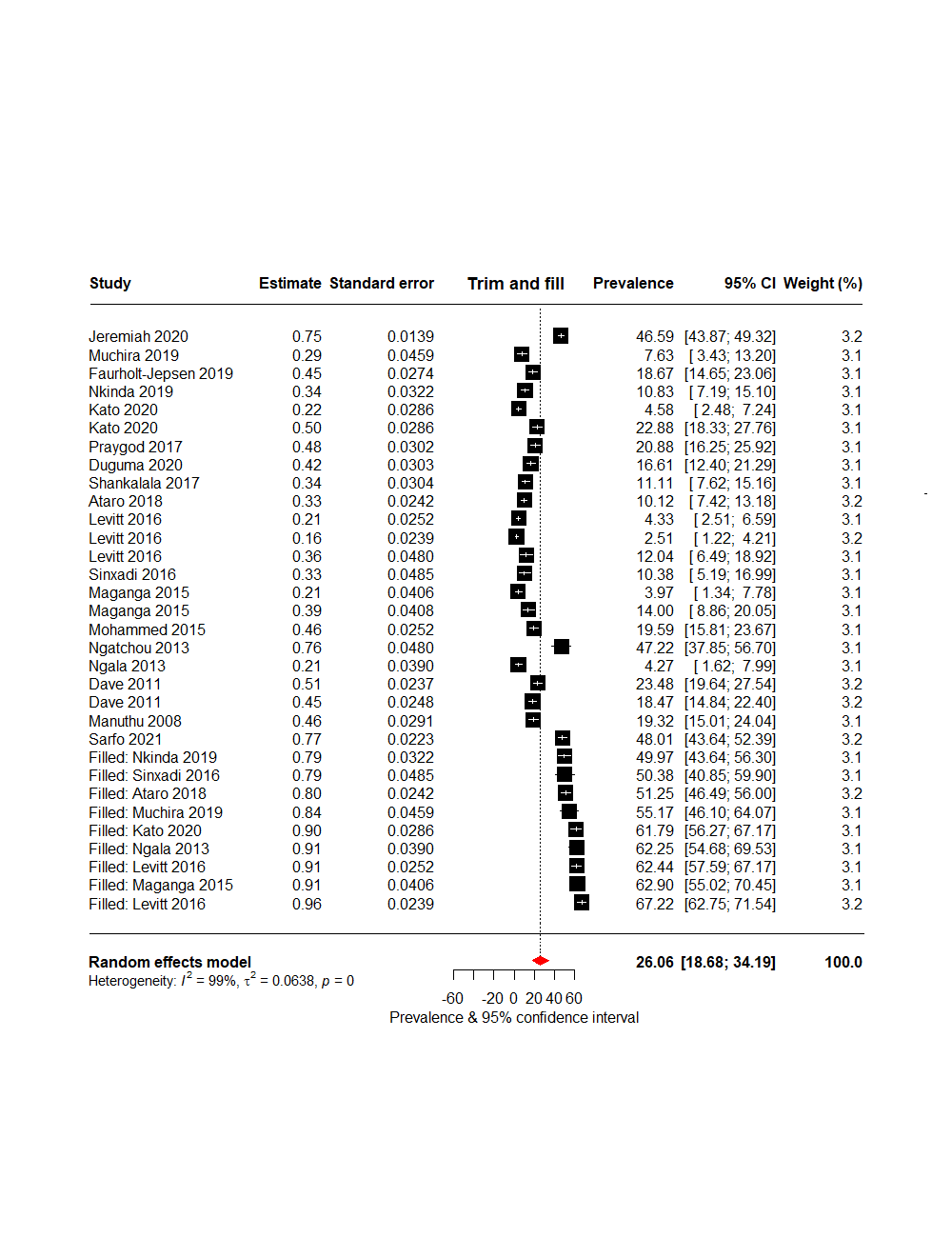

Supplement: Supplementary file 7 — Figure S7: Forest plot showing the pooled prevalence of pre‐diabetes in people living with HIV in studies in clinical settings, from the trim and fill analyses. [file JIA2-26-e26059-s004.tif]

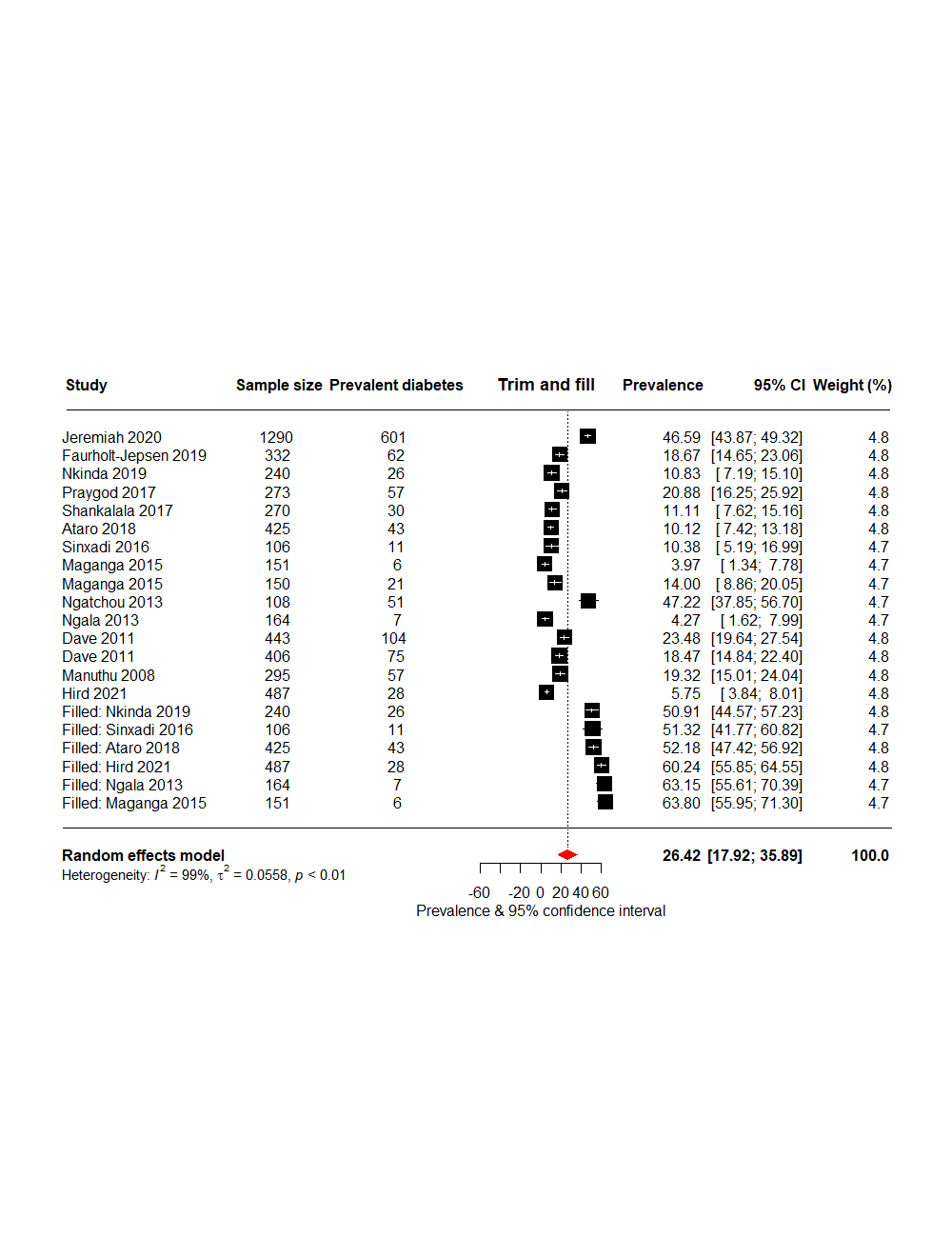

Supplement: Supplementary file 8 — Figure S8: Forest plot showing the pooled prevalence of pre‐diabetes in people living with HIV in studies in urban areas, from the trim and fill analyses. [file JIA2-26-e26059-s005.tif]
